# Validity of remote live stream video evaluation of the North Star Ambulatory Assessment in patients with Duchenne muscular dystrophy

**DOI:** 10.1371/journal.pone.0300700

**Published:** 2024-05-16

**Authors:** Linda P. Lowes, Lindsay N. Alfano, Megan A. Iammarino, Natalie F. Reash, Kathryn Giblin, Larry Hu, Lixi Yu, Shufang Wang, Rachel Salazar, Jerry R. Mendell

**Affiliations:** 1 Center for Gene Therapy, The Research Institute at Nationwide Children’s Hospital, Columbus, Ohio, United States of America; 2 Department of Pediatrics, The Ohio State University, Columbus, Ohio, United States of America; 3 Sarepta Therapeutics, Inc., Cambridge, Massachusetts, United States of America; University of Campania Luigi Vanvitelli: Universita degli Studi della Campania Luigi Vanvitelli, ITALY

## Abstract

Conducting functional assessments remotely can help alleviate the burden of in-person assessment on patients with Duchenne muscular dystrophy and their caregivers. The objective of this study was to evaluate whether scores from remote functional assessment of patients with Duchenne muscular dystrophy correspond to in-person scores on the same functional assessments. Remote live stream versus in-person scores on the North Star Ambulatory Assessment (including time [seconds] to complete the 10-meter walk/run and time to rise from the floor [supine to stand]) were assessed using statistical analyses, including intraclass correlation coefficient, and Pearson, Spearman, and Bland-Altman analyses. The remote and in-clinic assessments had to occur within 2 weeks of one another to be considered for this analysis. This analysis included patients with Duchenne muscular dystrophy, aged 4 to 7 years. Participants in this analysis received delandistrogene moxeparvovec (as part of SRP-9001-101 [Study 101; NCT03375164] or SRP-9001-102 [Study 102; NCT03769116]) or were randomized to receive placebo (in Part 1 of Study 102). This study evaluates score reproducibility between live stream remote scoring versus in-person functional assessments as determined by intraclass correlation coefficient, and Pearson, Spearman, and Bland-Altman analyses. The results showed that scores from remote functional assessment of patients with Duchenne muscular dystrophy strongly correlated with those obtained in person. These findings demonstrate congruence between live stream remote and in-person functional assessment and suggest that remote assessment has the potential to reduce the burden on a family by supplementing in-clinic visits.

## Introduction

Duchenne muscular dystrophy (DMD) is an X-linked, rare neuromuscular disease that is characterized by progressive muscle weakness and is caused by mutations in the *DMD* gene, which encodes the dystrophin protein [1]. The absence of functional dystrophin leads to a characteristic degeneration in skeletal and cardiac muscle [1–3]. Diagnosis of DMD is commonly delayed by 2.5 years after the time of symptom onset, with the average age at diagnosis of approximately 5 years [4–6]. From the time of initial diagnosis, the disease progresses rapidly, with onset of significant functional and motor impairments and the loss of ambulation occurring at 10–12 years [7, 8].

Corticosteroid therapy, the mainstay standard of care for DMD, aims to control symptoms and slow disease progression [9]. Long-term side effects associated with their use, however, include weight gain, behavioral changes, vertebral compression fractures secondary to osteoporosis, and cataracts [10–15]. Disease-modifying treatments address the underlying cause of disease, but are often only available for a subset of the patient population, as they are based on specific amenable mutations [16]. Delandistrogene moxeparvovec is a recombinant adeno-associated virus (rAAV)-based gene transfer therapy designed to compensate for the absence of functional dystrophin in DMD by delivering (via a single intravenous dose) a transgene encoding an engineered dystrophin protein that retains key functional domains of the wild-type protein [17]. It is currently approved in the USA, United Arab Emirates, and Qatar for the treatment of ambulatory pediatric patients aged 4 through 5 years with a confirmed mutation in the *DMD* gene [18, 19].

The reliable assessment of motor function is a foundational tool for measuring disease trajectory over time and evaluating whether an investigational treatment is efficacious in an intended patient population. Several established motor assessments are used in clinical trials of neuromuscular disease. In-person, onsite functional assessments conducted during clinical trials can be burdensome to patients with DMD and their families/caregivers. The need to travel to distant clinical trial assessment sites can require missed time at work or school, resulting in lost wages in addition to the base cost of travel to the site; this may skew trial participation towards more affluent families [20]. Moreover, the difficulties of in-clinic assessments were exacerbated by the restrictions and risks associated with the COVID-19 pandemic for patients, caregivers, and clinicians alike [21]. As a result, there exists a strong need for a complementary mode of reliably assessing motor function.

Guidance from the US Food and Drug Administration (FDA) and European Medicines Agency (EMA) on changes during the COVID-19 pandemic considered the safety, rights, and welfare of trial participants as paramount. Regulatory bodies additionally recommended considering whether trial visits could be conducted remotely, putting in place new processes, or modifying existing processes, in an attempt to accommodate participants and to maintain consistency with previous protocols [22, 23]. Conducting live stream functional assessments remotely, in the home of the patient or caregiver, rather than in clinic or at study sites, has the potential to improve patient experience, alleviate some of the burden of in-person assessments, and allow data collection to continue despite global travel restrictions. Additionally, in accordance with recent FDA guidance to encourage diversity and improve enrollment of participants from underrepresented populations in clinical trials, remote assessment may ease barriers to access, thereby improving the diversity of those enrolled [24, 25].

The North Star Ambulatory Assessment (NSAA) was specifically designed to measure motor function in DMD and is frequently used in both clinical and research settings [26]. The NSAA is a 17-item scale that grades performance of various motor skills, with a total score range of 0 to 34 (the highest score indicating the child was able to complete all tasks without modification or compensatory movement) (S1 Fig). Each skill on the NSAA is scored on a scale of 0 to 2, where 0 = unable to perform the skill; 1 = perform the skill with difficulty or compensatory movements; and 2 = able to perform the skill without compensatory movements [27]. In addition to scoring the performance of the child’s ability to run and rise from the floor, the time to complete these two items is recorded down to the 1/10 of a second. These numbers are not part of the NSAA total score but provide additional information (S1 Table). Considering recent regulatory guidelines and the post-COVID-19 landscape, it is important to establish the reliability and validity of using the NSAA during a live stream video meeting [28]. The NSAA was designed and validated as an in-person assessment and thus it may not translate exactly to the in-person assessments. In this study, the reproducibility and validity of live stream video remote functional assessment of patients with DMD by the NSAA, including two specific items with time components (supine to stand [Time to Rise] and 10-meter walk/run [10MWR]), were compared against in-person assessment by statistical analyses (intraclass correlation coefficient [ICC], Pearson, Spearman, and Bland-Altman).

## Materials and methods

### Participants

Participants in this analysis were a subset of the patients with DMD, aged 4–7 years, who were enrolled in ongoing studies of delandistrogene moxeparvovec: SRP-9001-101 (Study 101; NCT03375164; n = 4; enrollment began on 04 January 2018 and was completed on 25 April 2023) and SRP-9001-102 (Study 102; NCT03769116; n = 41; enrollment began on 05 December 2018 and was completed on 16 August 2023) at the Nationwide Children’s Hospital NCH, Columbus, Ohio, USA (S2 Table).

### Assessments

Assessment of the NSAA in Study 101 has been described previously [29, 30]. In Study 101, the NSAA was used as an exploratory functional endpoint and in Study 102, the NSAA was assessed as the primary endpoint, by measuring change from baseline in NSAA total score at week 48 (Part 1) [17]. Additionally, change from baseline in 10MWR and supine to stand scores from baseline to week 48 (Part 1) were assessed as secondary endpoints.

### Conducting live stream remote assessments

Remote assessments were conducted via the NCH telehealth system facilitated by Zoom video conferencing. The NCH physical therapist (PT) scored the NSAA in real time via a web meeting while the caregiver facilitated the assessment from their home. The PT instructed the caregiver to adjust the camera angle or provide additional instructions to the participant to ensure they could accurately score each item on the assessment. As per the NSAA manual, this could include requesting that the participant repeat an assessment. If the PT determined there was insufficient space to safely conduct the 10MWR in the patient’s home, the quality of the patient’s run was scored by watching the patient run shorter distances. The time was left blank as it was unable to be captured over 10 meters and a protocol deviation was noted. One important consideration for remotely timing supine to stand and the 10MWR test was having the in-home caregiver to say “go.” This eliminated the additional time lag seen when the PT said “go” remotely.

### Statistical analyses and considerations

The reproducibility of remote live-stream versus in-person scores on the NSAA, and the time to complete the 10MWR, and supine to stand was assessed using ICC (2.1, two-way random approach with absolute agreement) and Pearson, Spearman, and Bland-Altman analyses. To be considered for analysis, the remote and in-clinic assessments had to have been conducted within 2 weeks of one another. The remote and in-clinic NSAA assessments were considered comparable if total scores from these two visits were ≤3 points different. Differences in item scores (which have possible values of 0, 1, or 2) between remote and in-clinic assessments were calculated for each item of the NSAA.

### Ethics statement

Details of Study 101 and Study 102 have been published elsewhere [17, 30]. Study 101 (SRP-9001-101; NCT03375164) was approved by the institutional review board of Nationwide Children’s Hospital in Columbus, Ohio, USA. Signed informed consent was obtained from participants’ parents in compliance with the Code of Federal Regulations (Title 21, Part 50) and International Conference on Harmonization guidelines.

Study 102 (SRP-9001-102; NCT03769116) was approved by an internal review board at Sarepta Therapeutics, Inc., Cambridge, MA, United States in line with the Declaration of Helsinki and principles of Good Clinical Practice. The trial was approved by the institutional review boards of participating sites. Signed informed consent was obtained from participants’ parents, in compliance with the Code of Federal Regulations, Title 21, Part 50, and International Conference on Harmonization guidelines. Remote functional assessments were initiated during the COVID-19 pandemic, in accordance with FDA guidance.

## Results

### Overview of study data

During the COVID-19 pandemic, in accordance with FDA guidance, functional assessments conducted as part of ongoing clinical trials of delandistrogene moxeparvovec in patients with DMD were moved from on site to a remote setting, typically, the child’s home. These tests included the NSAA which includes two timed components (S2 Table). The mean number of days between assessments was 5.29. The total scores of the NSAA were highly correlated (ICC = 0.96). The degree of agreement for each individual item on the NSAA assessment were also calculated between remote and in-person scores and are shown in Table 1. Four items had perfect agreement: stand, stand up from chair, jump, and run. Although, still very similar, the highest degree of differences between in-clinic and remote assessments were seen on ascend and descend box step, stand on heels, and hop with an average point difference of 0.14, up to 0.24.

**Table 1:** Similarity score of remote versus in-person assessments for NSAA items.

Item	Number of pairs with perfect agreement N = 21	Average point difference	Range of differences (possible NSAA item scores: 0, 1, 2)
Stand	21 (100%)	0.00	0–0
Walk	20 (95%)	0.05	0–1
Stand up from chair	21 (100%)	0.00	0–0
Stand on one leg–right	20 (95%)	0.05	0–1
Stand on one leg–left	20 (95%)	0.05	0–1
Climb box step–right	16 (76%)	0.24	0–1
Climb box step–left	17 (81%)	0.19	0–1
Descend box step–right	18 (86%)	0.14	0–1
Descend box step–left	17 (81%)	0.19	0–1
Gets to sitting	20 (95%)	0.05	0–1
Rise from floor	20 (95%)	0.05	0–1
Lifts head	20 (95%)	0.05	0–1
Stands on heels	17 (81%)	0.19	0–1
Jump	21(100%)	0.00	0–0
Hop right leg	17 (81%)	0.19	0–1
Hop left leg	17 (81%)	0.19	0–1
Run (10m)	21(100%)	0.00	0–0

NSAA items were assessed for 21 in-clinic or remote sessions. A similarity score was calculated as the difference in scores between in-clinic and remote sessions. NSAA, North Star Ambulatory Assessment.

### Similarity between remote and in-clinic assessments

**Fig 1:**
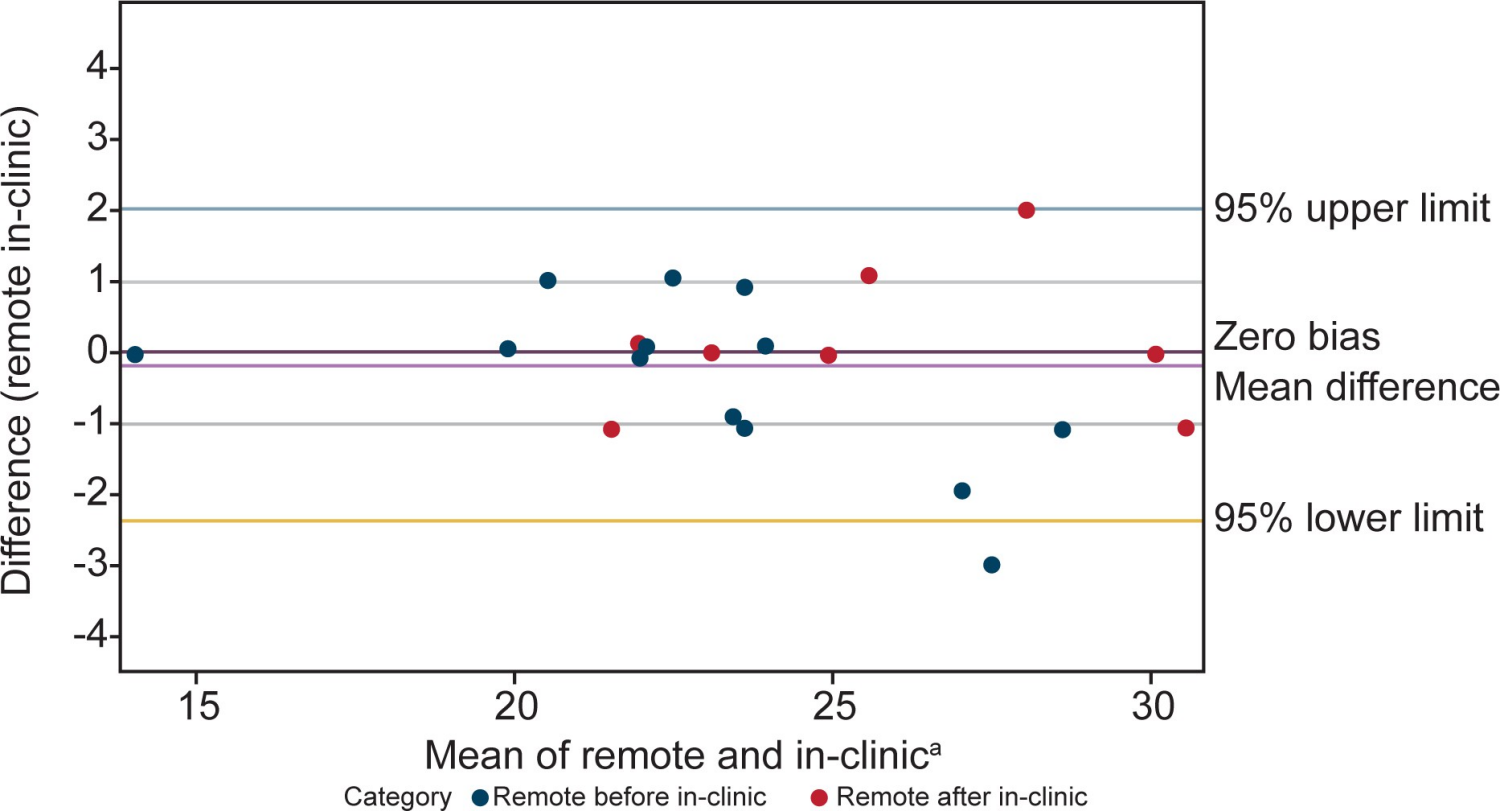
Bland-Altman analysis plot of remote and in-clinic assessments shows relative agreement between the two modalities for the NSAA. Measurements closer to the center line of zero bias mean difference indicate higher levels of agreement. ^a^X-axis is time in seconds. NSAA, North Star Ambulatory Assessment.

**Fig 2:**
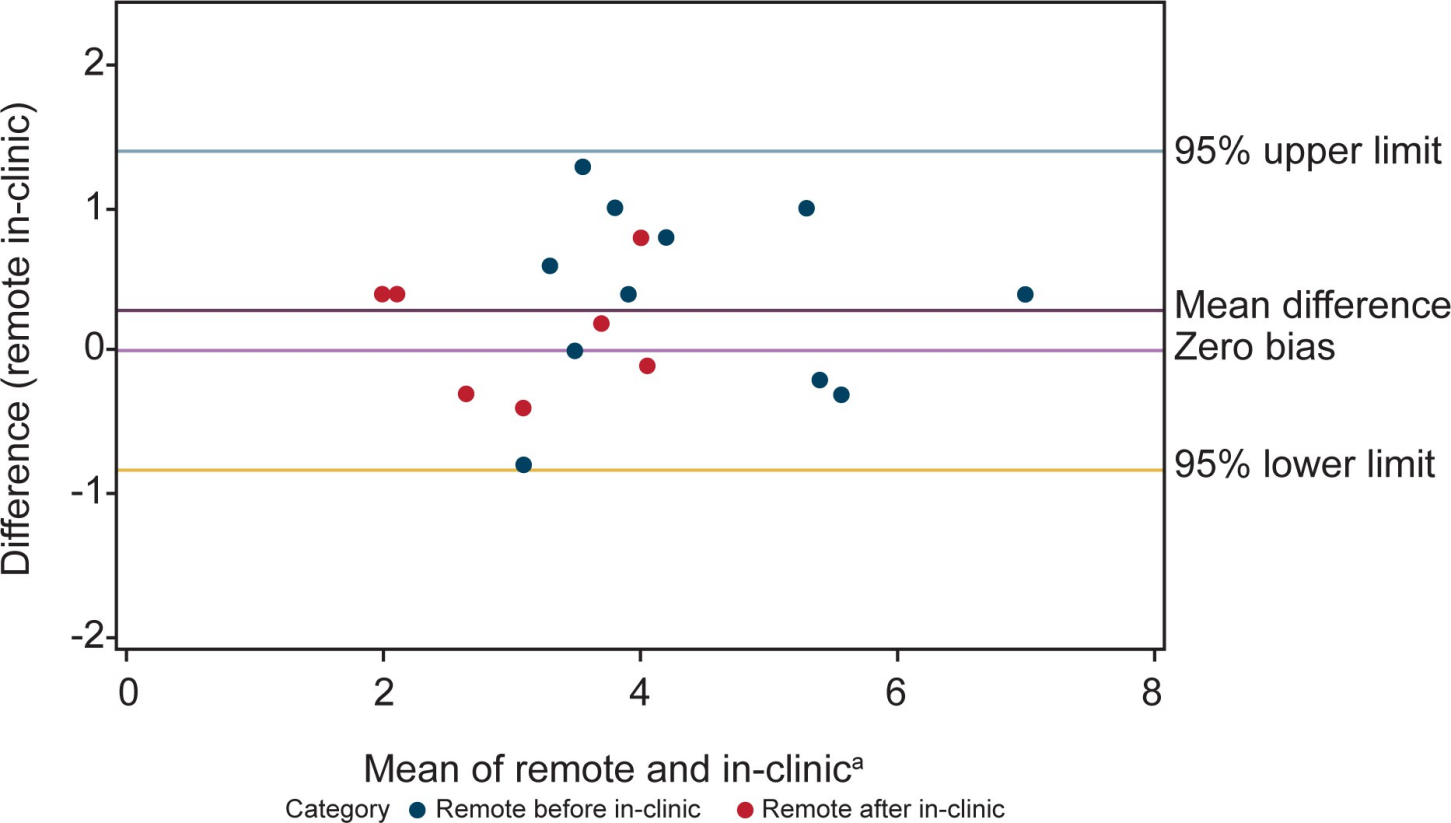
Bland-Altman analysis plot of remote and in-clinic assessments shows relative agreement between the two modalities for the supine to stand assessment. Measurements closer to the center line of zero bias mean difference indicate higher levels of agreement. ^a^X-axis is time in seconds.

**Fig 3:**
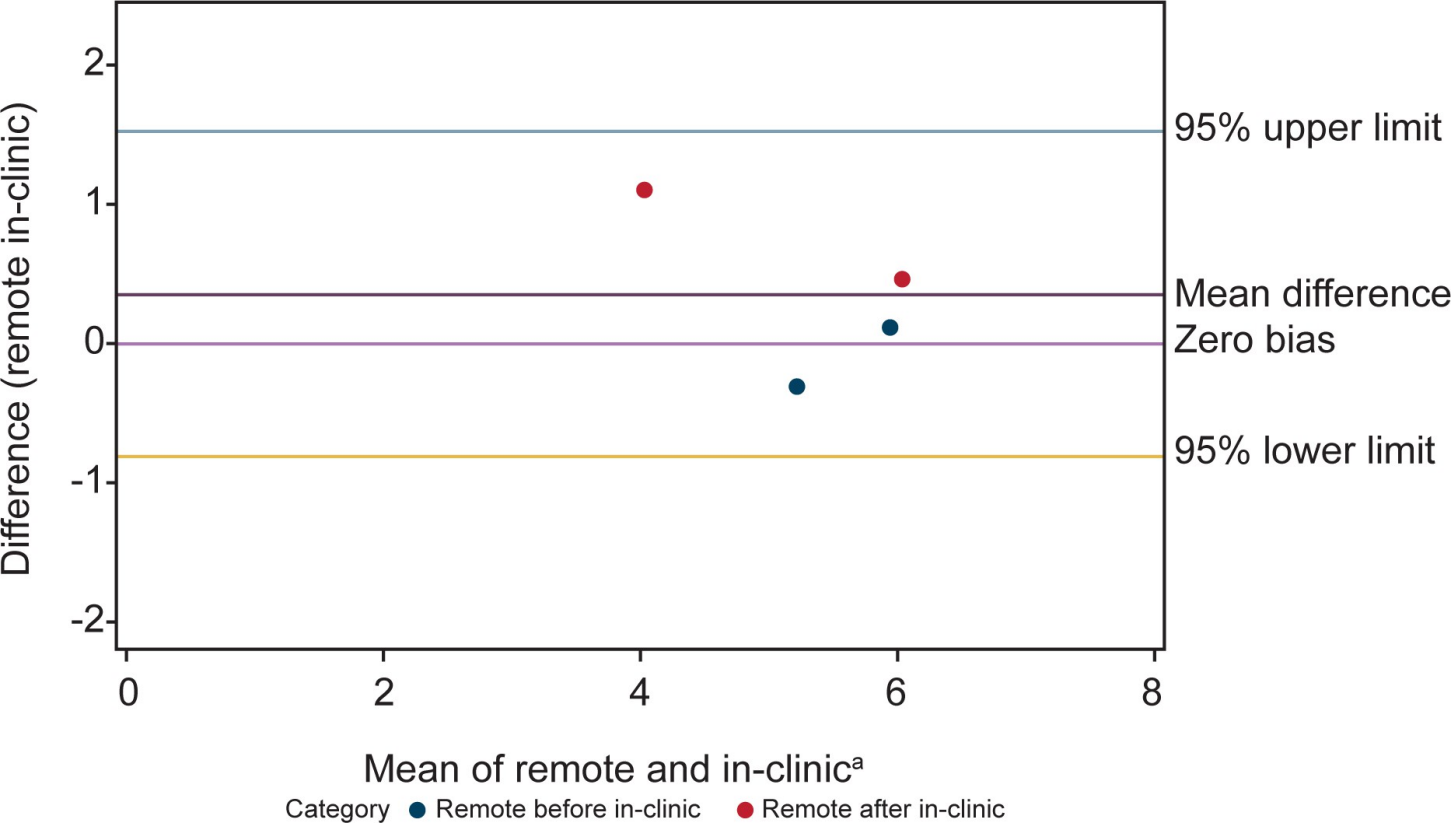
Bland-Altman analysis plot of remote and in-clinic assessments shows relative agreement between the two modalities for the 10MWR assessment. Measurements closer to the center line of zero bias mean difference indicate higher levels of agreement. ^a^X-axis is time in seconds. 10MWR, 10-meter Walk/Run.

Bland-Altman analysis showed concordance between scores from NSAA assessments conducted at home and in-person. There were 18 patients with comparable assessments. Of the 18 patients, some had more than one pair of comparable assessments (conducted within 2 weeks of one another, with total scores ≤3 points different) resulting in a total of 21 remote and in-clinic assessment pairs being included in the Bland-Altman analysis (Fig 1). Similarly, agreement was seen in Bland-Altman analyses between the time to complete the supine to stand (n = 18 remote and in-clinic assessment pairs) and 10MWR (n = 4 remote and in-clinic assessment pairs) from remote and in-person assessment of timed function tests (Figs 2 and 3).

### Results of timed function tests assessed remotely correlated with those conducted in person

**Fig 4:**
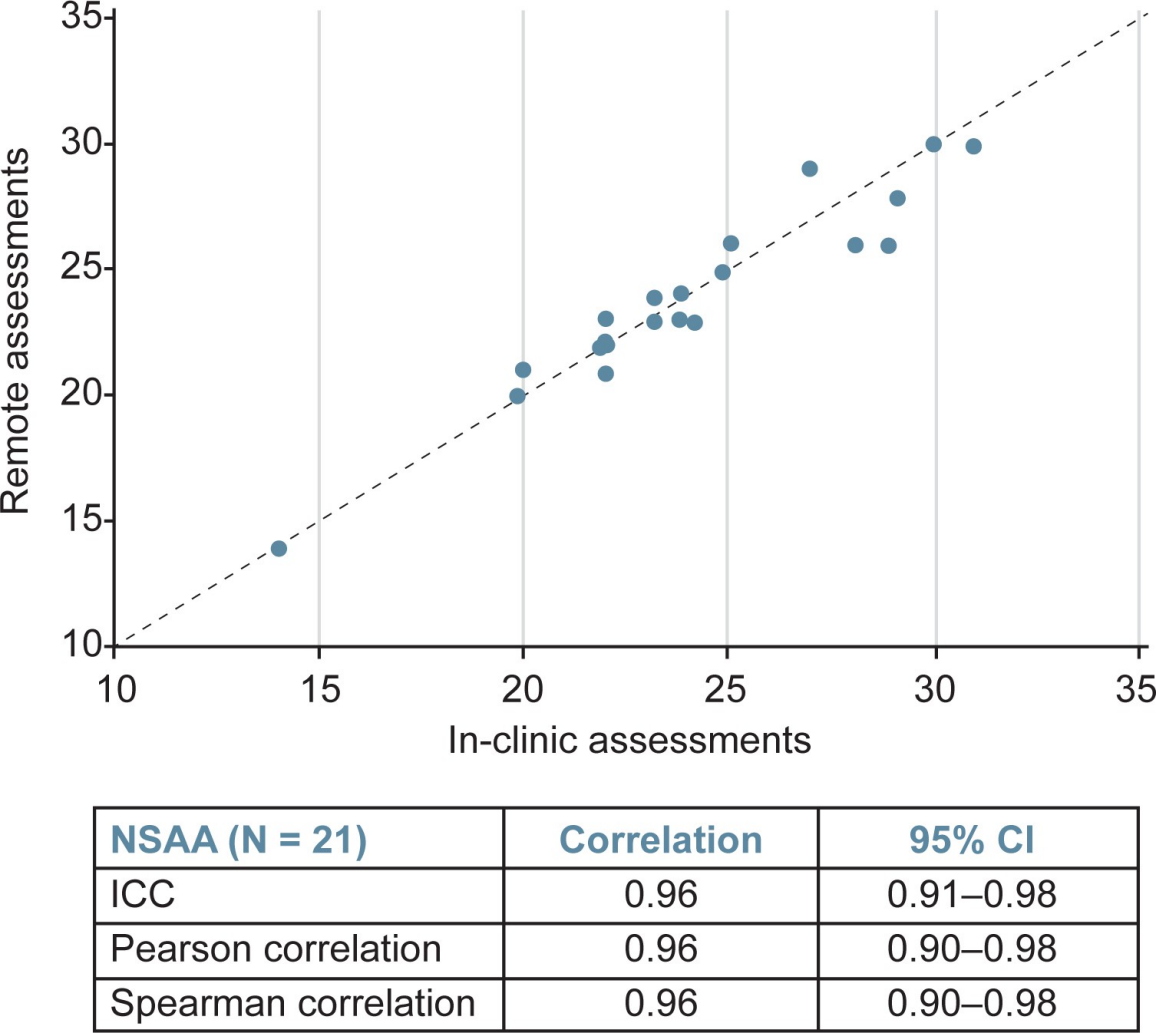
Correlation analysis of remote and in-clinic assessments demonstrates strong correlation between both modalities for the NSAA. CI, confidence interval; ICC, intraclass correlation coefficient; NSAA, North Star Ambulatory Assessment.

**Fig 5:**
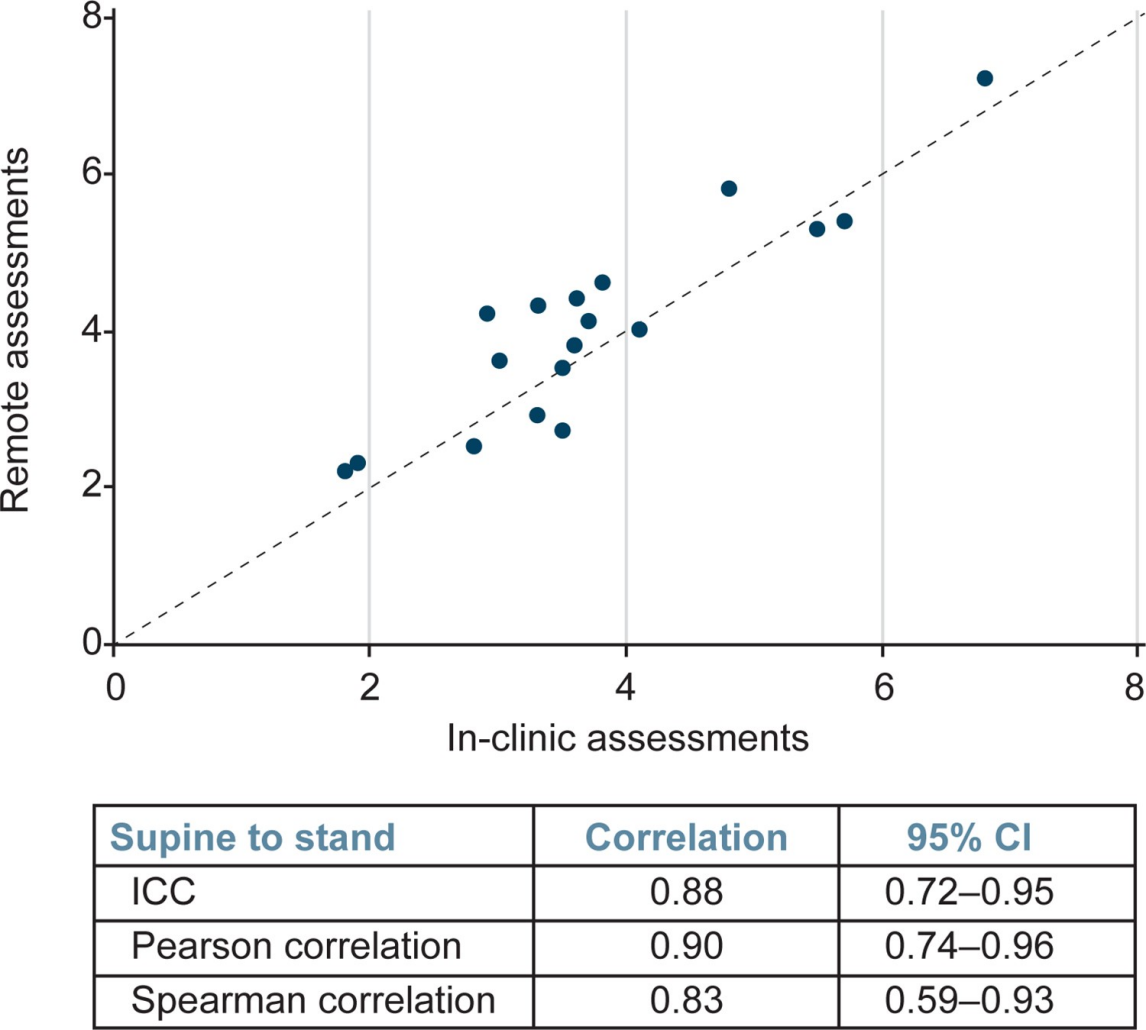
Correlation analysis of remote and in-clinic assessments demonstrates strong correlation between both modalities for the supine to stand assessment. CI, confidence interval; ICC, intraclass correlation coefficient.

**Fig 6:**
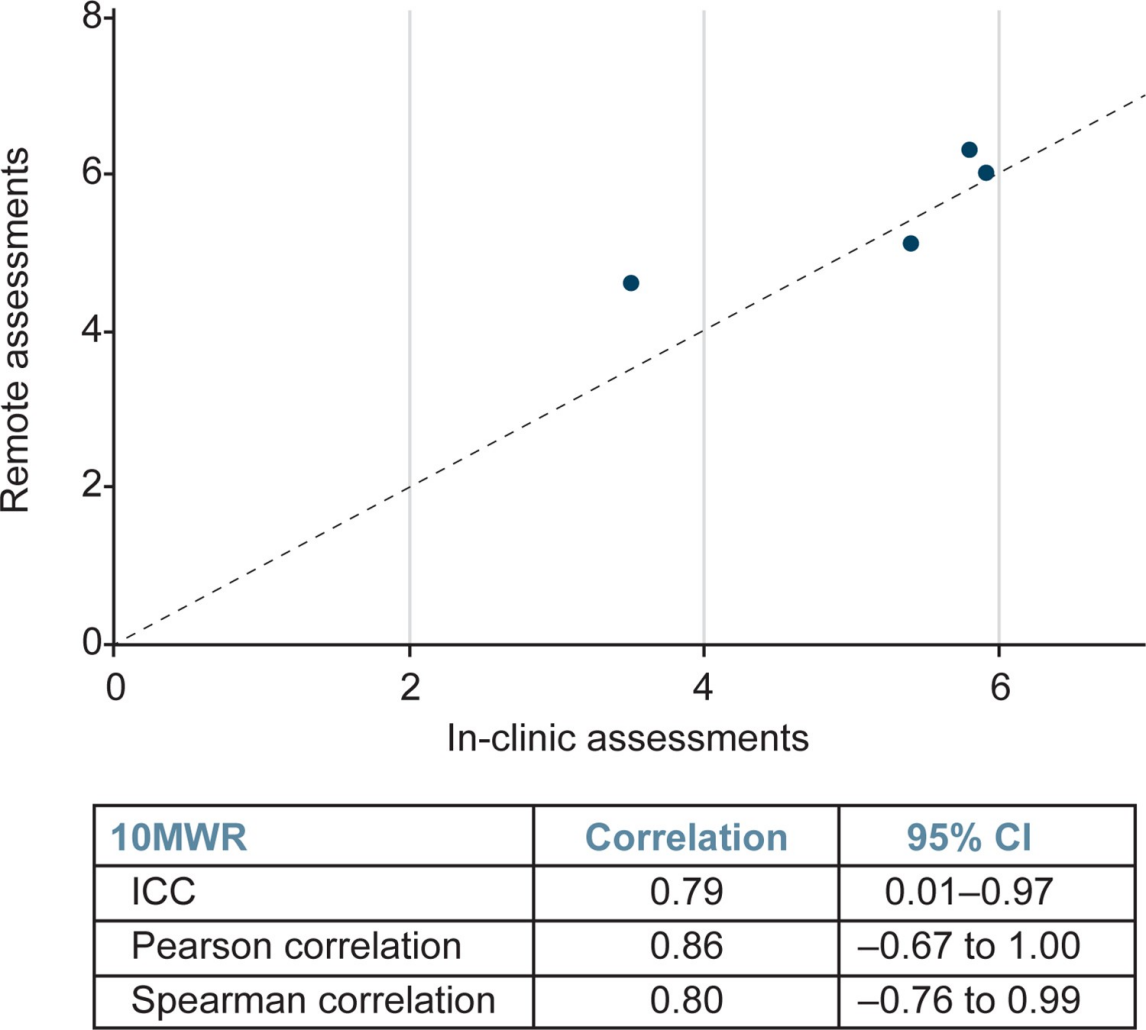
Correlation analysis of remote and in-clinic assessments demonstrates strong correlation between both modalities for the 10MWR assessment. 10MWR, 10-meter Walk/Run; CI, confidence interval; ICC, intraclass correlation coefficient.

Further, correlation analyses demonstrated that NSAA scores (n = 21) from remote assessment strongly correlated with those attained within 2 weeks via in-person assessment (Fig 4), with the ICC demonstrating correlation of 0.96 (95% confidence interval [CI]: 0.91–0.98), the Pearson correlation demonstrating correlation of 0.96 (95% CI: 0.90–0.98), and the Spearman correlation demonstrating correlation of 0.96 (95% CI: 0.90–0.98). The time to complete the supine to stand (n = 18) and 10MWR (n = 4) assessed remotely also correlated well with times attained in person (Figs 5 and 6). The ICC demonstrated a correlation between in clinic and remote assessment of supine to stand of 0.88 (95% CI: 0.72–0.95), while the Pearson correlation was 0.90 (95% CI: 0.74–0.96) and the Spearman correlation was 0.83 (95% CI: 0.59–0.93). The intraclass correlation coefficient demonstrated a correlation between in-clinic and remote assessment on the 10MWR of 0.79 (95% CI: 0.01–0.97), while the Pearson correlation was 0.86 (95% CI: –0.67 to 1.00) and the Spearman correlation was 0.80 (95% CI: –0.76 to 0.99).

## Discussion

Remote assessment of clinical trial outcome measures has long been recognized as an unmet need—especially in rare diseases like DMD, where travel to clinic can be a significant burden to patients and caregivers [31]. The COVID-19 pandemic accelerated the demand for remote assessment in clinical trials. Both the FDA and the EMA issued guidance on changes during the pandemic to protect patients and facilitate continued trial execution while maintaining good clinical practice standards [22, 23]. Both agencies strongly emphasize the importance of ensuring study participant safety, rights, and welfare. Additionally, the agencies promoted consideration of remote visits, given that in-person visits may place additional risk on participating patients. The FDA guidance provides detailed recommendations for remote assessments and indicates that all sponsors should provide rationale and feasibility of such assessments, as well as the methods to ensure participant compliance and data collection consistency [32].

In this study, Bland-Altman analyses showed agreement between remote and in-person assessments of the NSAA, supine to stand, and 10MWR—regardless of whether remote or in-person assessment occurred first. Remote evaluation of the NSAA, as compared to the in-person NSAA, also demonstrated strong correlation. Specifically, the NSAA showed an excellent ICC of 0.96 (95% CI: 0.91–0.98), while the Pearson correlation was 0.96 (95% CI: 0.90–0.98) and the Spearman correlation was 0.96 (95% CI: 0.90–0.98). Testing young children can sometimes be challenging even for a physical therapist with pediatric experience. Perhaps surprisingly, the younger 4- to 5-year-old boys had similar stability across sites comparable to the older 6- to 7-year-old boys. The median difference for both groups was 0 and the averages were also similar (0.75 for 4- to 5-year-old boys, and 0.78 for 6- to 7-year-old boys).

Though some variability was observed in results derived from remote versus in-person supine to stand and 10MWR analyses, this was not found to be significant, and the scores were shown to be concordant. The least consistent items between remote and in-clinic visits were climb box step, descend box step, stand on heels, and hop. The most consistent items were stand, stand up from chair, jump, and run. In this regard, a study that assessed the reliability and validity of in-clinic assessment of NSAA in ambulant boys with DMD in Brazil showed the largest difference in stands on right and left leg, jumps, and walking [33]. Overall, the intra-rater in-clinic total score reliability reported by Okama et al. (ICC = 0.98) was only slightly higher than what we report (ICC = 0.96), suggesting that the differences between assessments performed onsite or remotely are similar to what one might expect due to variability of the score between days, rather than to setting differences [33].

In our study we noted that variability between scores on in-person versus remote assessments appeared to increase as performance on the NSAA increased. This is likely due to the higher number of items that the patient could functionally complete.

The findings from this study suggest that remote assessment of function in patients with DMD is not statistically or clinically different from the accepted “gold standard” of in-person assessment, and that remote evaluation is a statistically valid means of performing the NSAA in patients with DMD. It is important to remember, however, that this is a single-center study, limited by a small sample size. Further, bias from recall of performance by the evaluator is an additional potential limitation of this study, given that most paired assessments evaluated occurred within 1 week of each other. Thus, the interpretation of the second assessment could have been influenced by the first. Ideally, a larger, more diverse patient-population, inclusive of more clinical trial sites, would help determine whether the present findings are reproducible in a more diverse and representative cohort of individuals with DMD.

Another recent publication also clinically tested the question of whether two evaluators had high inter-rater reliability in scoring when watching the same video of an assessment recorded in the families’ homes [28]. While this study by Emery et al. hinted at the promise of conducting the NSAA remotely, this group did not have a contemporaneous in-person comparator, but rather compared the performance to previous clinic visits 6 and 12 months earlier and determined concordance by estimating a 2.2-point decline over 12 months, and then compared this extrapolated measure to the result of the videotaped assessment. In addition, assessing inter-rater reliability exclusively by video-assessed scores introduces a variable not encountered with live versus video-assessed scores.

The findings from the present study dbute to the accumulating evidence suggesting that remote assessment is feasible in clinical trial settings. Alongside this growing evidence, recommendations are emerging regarding the legal, regulatory, and practical challenges associated with remote assessment in decentralized clinical trials conducting these assessments in the clinic [31]. For example, a public-private partnership, the Clinical Trials Transformation Initiative (CTTI), launched the Decentralized Clinical Trials Project to provide guidance on factors such as protocol design, telemedicine use, mobile healthcare providers, medical supply chain, investigator delegation and oversight, and remote-administration safety considerations [31]. Additionally, other publications, such as James et al., have offered direction on methodological considerations specific to remote assessment, establishing initial guidelines for the suitability and feasibility of performing remote evaluations of commonly used endpoints in clinical trials of neuromuscular diseases [21]. These considerations were established by expert clinical evaluators and physical therapists, and assessed multiple factors, including safety, standardization of equipment and the home testing environment, accuracy and reliability of scoring via live-feed, data security and privacy regulations, accurate patient identification, and family and patient consent.

As evidenced above, telehealth technology has become more widespread and continues to show promise for reducing patient burden, increasing access and clinical trial diversity. As such, there have been efforts to initiate and validate a variety of remotely administered clinical outcome assessments, in addition to those used for neuromuscular disease. These include assessments in neurological indications, as well as conditions and/or studies spanning all ages—children, adolescents, adults, and the elderly. For example, the Wechsler Intelligence Scales for Children, Fifth Edition (WISC-V) [34], child speech and language evaluation [35], and the Alzheimer’s Disease Assessment Scale cognitive subscale (ADAS-cog) have both been tested for congruence of results attained in-person versus at home [36]. These studies have demonstrated high agreement between remote and in-person assessment, using similar statistical methodologies as were employed here.

Overall, this methodology for assessing clinical trial outcome will improve over time. Our study demonstrates that remote functional assessments can be achieved, even for assessment of a complex task like the NSAA, especially when alternatives are limited as during the COVID-19 pandemic. These data add to the promising body of literature showing the potential promise of such approaches both in clinical trial and real-world practice.

## Supporting information

S1 Fig NSAA is a composite endpoint evaluating physical function across 17 tests with increasing difficulty. NSAA, North Star Ambulatory Assessment.
(PDF)

S1 Table Overview of the items on the NSAA. NSAA, North Star Ambulatory Assessment.
(PDF)

S2 Table Overview of participants included in this analysis. ^a^These patients were to receive delandistrogene moxeparvovec in Part 2 of Study 102. As all remote assessment data are from Part 1 of Study 102, these patients did not receive delandistrogene moxeparvovec treatment. NSAA, North Star Ambulatory Assessment.
(PDF)

S3 Table Individual NSAA item scores for remote and in-clinic assessments. NSAA, North Star Ambulatory Assessment.
(XLSX)

S4 Table Individual NSAA total score, time to rise from the floor (supine to stand), and 10MWR for remote and in-clinic assessments. 10MWR, 10-meter walk/run; NSAA, North Star Ambulatory Assessment.
(XLSX)

S1 Protocol Clinical study protocol. (PDF)

S1 File Consent to participate in a clinical research study. (DOCX)

S1 Data (XLSX)

S2 Data (XLSX)
