# Supplementary material for: Validity of remote live stream video evaluation of the North Star Ambulatory Assessment in patients with Duchenne muscular dystrophy
Source: PLoS One. 2024 May 16;19(5):e0300700. doi: 10.1371/journal.pone.0300700 (PMC11098514; doi:10.1371/journal.pone.0300700)
Supplement: S1 Fig — NSAA, North Star Ambulatory Assessment. (PDF) [file pone.0300700.s001.pdf]

S1 Fig. NSAA is a composite endpoint evaluating physical function across 17 tests with increasing difficulty

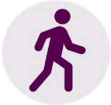

Score	Ability
2	Perform
1	Perform with compensatory movements
0	Unable to Perform

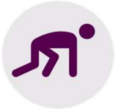

Assessment	Relevant activities of daily living
Items 14–17: Jump, hop, run	Playing, accessing sports, keeping up socially and physically with peers, skipping, hopscotch
Item 13: Stand on heels	Walking on uneven or hilly ground, cycling more easily, getting out of chair and stepping more easily
Item 11: Rise from floor	Getting up after falling down, sitting on floor with classmates without needing help to get up
Item 10: Gets to sitting	Sitting up in bed, assuming a safer position if fall occurs
Items 6–9: Climb on and off box step	Independent outdoor mobility (curbs in particular)
Items 4 & 5: Stand on one leg	Kicking a ball, stepping off a curb, putting on pants, shoes and socks while standing
Item 3: Stand up from chair	Moving from class to class, using a toilet, getting out of bed or a car
Item 2: Walk	Participating in peer related activities, mobility in the home, school and community
Item 1: Stand	Standing to pee, groom, prepare meals, or access high items like elevator buttons, light switches and cupboards

NSAA, North Star Ambulatory Assessment.
