# Supplementary material for: Validity of remote live stream video evaluation of the North Star Ambulatory Assessment in patients with Duchenne muscular dystrophy
Source: PLoS One. 2024 May 16;19(5):e0300700. doi: 10.1371/journal.pone.0300700 (PMC11098514; doi:10.1371/journal.pone.0300700)
Supplement: S1 Table — NSAA, North Star Ambulatory Assessment. (PDF) [file pone.0300700.s002.pdf]

S1 Table. Overview of the items on the NSAA

Assessment	Starting Position	Instruction	Remote	Case-by-case
1: Standing	Patient’s feet must be about 4 inches (about a hands width) apart and patient’s toes must be facing forward.	Stand straight and still for 3 seconds.	✓	
2: Walk	Patient standing will walk back and forth in front of the camera. Camera must be on the side view and then on the front view.	Walk from A to B.	✓	Requires ~8 feet clear floor
3: Stand up from chair	Patient will sit on an adjustable height bench where patient’s hips and knees are at a 90º angle from the floor. Patient’s feet should be about hip width apart and toes pointing forward. Patient will first try to stand up with his arms crossed over his chest.	Stand up from the chair keeping arms folded.	✓	Requires adjustable height bench
4 & 5: Stand on one leg, first right and then left	Patient will be standing.	Stand on right leg for as long as possible with arms at sides. Count “And 1, and 2, and 3….” Repeat with left leg.	✓	
6 & 7: Climb box step, first right and then left	Patient will stand in front of the box step, 10 inches away.	Step onto the top of the box with right leg. Repeat with left leg.	✓	Requires box step
8 & 9: Descend box step, first right leg and then left leg	Patient will stand on box step with toes lined up close to the edge.	Step down from the box with right leg. Repeat with left leg.	✓	Requires box step

10: Gets to sitting	Patient will lie on the floor with legs straight and arms by sides.	Lying into sitting position, using only one hand, push yourself up.	✓	
11: Rise from floor	Patient will start in supine position with arms by sides, legs straight.	Once instructed, get up as fast as possible and stand with arms by sides using as little support as possible.	✓	
12: Lifts head	Patient will lie on the floor with arms across chest. Do not use a pillow or a mat.	Lift head and touch chin to chest, keeping arms folded.	✓	
13: Stands on heels	Patient will stand on the floor. No shoes to be worn.	Stand on heels. Count ‘And 1, and 2, and 3….”	✓	
14: Jump	Patient will stand on the floor, feet comfortably close together.	Record greatest height that can be achieved while jumping.	✓	
15 & 16: Hop on right leg and then left leg	Patient will stand on the floor on right leg, then left leg.	Hop on right leg, then on left leg.	✓	
17: Run (10 meters)	Patient will be standing.	Put a tape line down for a starting line and put another line 10 meters away using the tape measure provided by Sarepta. Patient should position himself so that patient’s toes are behind the starting line. Patient will run down and not stop until he gets all the way to past the other side. Camera will be positioned by the finish line.	✓	Requires 10 meters (32.8 feet) clear floor space or outside space. If there is not enough space, the CE will observe the patient run back and forth in a smaller space.
