# Supplementary material for: Validity of remote live stream video evaluation of the North Star Ambulatory Assessment in patients with Duchenne muscular dystrophy
Source: PLoS One. 2024 May 16;19(5):e0300700. doi: 10.1371/journal.pone.0300700 (PMC11098514; doi:10.1371/journal.pone.0300700)
Supplement: S2 Table — aThese patients were to receive delandistrogene moxeparvovec in Part 2 of Study 102. As all remote assessment data are from Part 1 of Study 102, these patients did not receive delandistrogene moxeparvovec treatment. NSAA, North Star Ambulatory Assessment. (PDF) [file pone.0300700.s003.pdf]

S2 Table. Overview of participants included in this analysis

NSAA	Study 101	Study 102		Both
	Delandistrogene moxeparvovec N = 4	Assigned to received delandistrogene moxeparvovec in Part 1 (N = 20)	Assigned to receive placebo in Part 1 (N = 21)[a]	Total N = 45
**Number of remote assessments**	5	31	22	58
**Number of patients with remote assessments**	2	12	9	23
**Number of patients with remote and in-clinic assessments (within 2 weeks of one another)**	1	9	8	18
Number of patients with only one pair of remote and in-clinic assessments (within 2 weeks of one another)	0	8	7	15
Number of patients with more than one pair of remote and in-clinic assessments (within 2 weeks of one another)	1	1	1	3
**Comparable remote and in-clinic assessments**				
**Number of patients with comparable assessments**	1	9	8	18
**Number of instances (pairs of assessments)**	2	10	9	21

[a]These patients were to receive delandistrogene moxeparvovec in Part 2 of Study 102. As all remote assessment data are from Part 1 of Study 102, these patients did not receive delandistrogene moxeparvovec treatment.

NSAA, North Star Ambulatory Assessment.
