# Supplementary material for: Validity of remote live stream video evaluation of the North Star Ambulatory Assessment in patients with Duchenne muscular dystrophy
Source: PLoS One. 2024 May 16;19(5):e0300700. doi: 10.1371/journal.pone.0300700 (PMC11098514; doi:10.1371/journal.pone.0300700)
Supplement: S1 Protocol — (PDF) [file pone.0300700.s006.pdf]

CLINICAL STUDY PROTOCOL

**DRUG:**	SRP-9001 (formerly referred to as rAAVrh74.MHCK7.microdystrophin)
**STUDY NUMBER:**	SRP-9001-102
**STUDY TITLE:**	A Multicenter, Randomized, Double-Blind, Placebo-Controlled Trial for Duchenne Muscular Dystrophy Using SRP-9001
**IND NUMBER:**	17763
**SPONSOR:**	Sarepta Therapeutics, Inc.<br>215 First Street<br>Cambridge, MA 02142 USA<br>Phone: +1-617-274-4000
**CURRENT VERSION DATE:**	Version 8 (Amendment 7), 27 August 2020
**REPLACES VERSION DATE:**	Version 7 (Amendment 6), 18 May 2020<br>Version 6 (Amendment 5), 25 September 2019<br>Version 5 (Amendment 4), 02 July 2019<br>Version 4 (Amendment 3), 04 June 2019<br>Version 3 (Amendment 2), 26 February 2019<br>Version 2 (Amendment 1), 09 November 2018<br>Version 1 (Amendment 0), 13 April 2018

CONFIDENTIALITY STATEMENT

The information contained in this document, is the property of the Sponsor and is confidential. This information may not be disclosed, reproduced or distributed to anyone other than personnel directly involved in the conduct of the study and in response to a relevant Institutional Review Board/Independent Ethics Committee and Review by a Regulatory Authority as required by the applicable laws and regulations, without the written authorization of the Sponsor, except to the extent necessary to obtain written informed consent from those individuals to whom the drug may be administered. These restrictions will continue to apply after the study has closed.

SIGNATURE PAGE

**Protocol Title:**	A Multicenter, Randomized, Double-Blind, Placebo-Controlled Trial for Duchenne Muscular Dystrophy Using SRP-9001
**Study No:**	SRP-9001-102
**Current Version Date:**	Amendment 7 (Version 8), 27 August 2020

This study protocol (Version 8, Amendment 7 dated 27 August 2020) was subject to critical review and has been approved by the appropriate protocol review committee of Sarepta Therapeutics, Inc. The information contained in this protocol is consistent with:

- The current benefit-risk evaluation of the investigational products (IPs).
- The ethical and scientific principles governing clinical research as set out in the Declaration of Helsinki, and principles of Good Clinical Practice (GCP) as described in 21 Code of Federal Regulations (CFR) parts 50, 54, 56, and 312 and the European Clinical Trial Directive 2001/20/EC.

The Investigator will be supplied with details of any significant or new findings, including adverse events (AEs), relating to treatment with the IPs.

[redacted]

Electronically signed by: [redacted]
Reason: I approve this document.
Date: Aug 28, 2020 11:41 EDT

28-Aug-2020

[redacted]

Date

INVESTIGATOR'S AGREEMENT

I have received and read the Investigator's Brochure for SRP-9001. I have read the SRP-9001-102 Study Protocol (Version 8, Amendment 7 dated 27 August 2020) and agree to conduct the study as outlined. I agree to maintain the confidentiality of all information received or developed about this protocol.

____

Printed Name of Investigator

____

Signature of Investigator

____

Date

TABLE OF CONTENTS

CLINICAL STUDY PROTOCOL 1
SIGNATURE PAGE 2
INVESTIGATOR'S AGREEMENT 3
TABLE OF CONTENTS 4
LIST OF TABLES 8
LIST OF ABBREVIATIONS AND DEFINITIONS OF TERMS 9
1. PROTOCOL SUMMARY 11
1.1. Synopsis 11
1.2. Schema 15
1.3. Schedule of Activities 16
2. INTRODUCTION 21
2.1. Study Rationale 21
2.2. Background 21
2.3. Benefit/Risk Assessment 21
3. STUDY DESIGN 23
3.1. Overall Design 23
3.1.1. Screening/Baseline Period 23
3.1.2. Treatment Period 23
3.1.2.1. Part 1 23
3.1.2.2. Part 2 24
3.1.2.3. Part 3 25
3.2. Scientific Rationale for Study Design 26
3.3. [redacted] 26
3.4. End of Study Definition 27
4. STUDY POPULATION 28
4.1. Inclusion Criteria 28
4.2. Exclusion Criteria 28
4.3. Lifestyle Considerations 29
4.4. Screen Failures 29
5. STUDY INTERVENTION 30
5.1. Study Intervention(s) Administered 30

5.1.1. Pre-infusion Immunosuppressants 30
5.1.2. Post-infusion Immunosuppressants 30
5.1.3. Intravenous Infusion (Day 1, Part 1 and Part 2) 31
5.1.4. Post Infusion Monitoring 31
5.1.4.1. Immediately Following Infusion 31
5.1.4.2. [redacted] 31
5.2. Preparation/Handling/Storage/Accountability 31
5.3. Measures to Minimize Bias: Randomization and Blinding 31
5.3.1. Establish Patient Identification Number 31
5.3.2. Assignment to a Treatment Group 31
5.3.3. Randomization 32
5.3.4. Blinding/Unblinding 32
5.4. Concomitant Therapy 33
5.4.1. Rescue Medicine 33
5.5. Dose Modification 33
5.5.1. Stopping Rules 33
5.5.2. Dosing Schedule 34
5.6. Intervention after the End of the Study 34
6. STUDY ASSESSMENTS AND PROCEDURES 35
6.1. Efficacy Assessments 35
6.1.1. Muscle Biopsy 35
6.1.2. Immunofluorescence Fiber Intensity / Immunofluorescence PDPF 35
6.1.3. [redacted] 35
6.1.4. [redacted] 35
6.1.5. Physical Functional Assessments 36
6.1.5.1. Time to Rise from the Floor 36
6.1.5.2. Ascend 4 Steps 36
6.1.5.3. North Star Ambulatory Assessment 36
6.1.5.4. 10-meter Timed Test 36
6.1.5.5. 100-meter Timed Test 36
6.2. Safety Assessments 36
6.2.1. Physical Examinations 36
6.2.2. Vital Signs 37

6.2.3. Electrocardiograms and Echocardiograms 37
6.2.4. Clinical Safety Laboratory Assessments 37
6.2.5. Safety Monitoring 39
6.2.5.1. Safety Monitoring for Liver Chemistry Tests 39
6.2.5.2. Safety Monitoring for Hypersensitivity and Complement-Mediated Reactions 39
6.2.5.3. Safety Monitoring for Platelet Count Results 40
6.2.5.4. Safety Monitoring for Rhabdomyolysis 41
6.2.6. Immunogenicity Assessment 41
6.2.7. [redacted] 42
6.2.8. [redacted] 42
6.3. Adverse Events and Serious Adverse Events 42
6.3.1. Collection of Adverse Events 42
6.3.2. Definition of Adverse Events 42
6.3.2.1. Adverse Event 42
6.3.2.2. Serious Adverse Events 43
6.3.2.3. Adverse Events of Special Interest 43
6.3.3. Classification of Adverse Events 43
6.3.3.1. Relationship to Investigational Product 43
6.3.3.2. Relationship to Study Procedures 44
6.3.3.3. Relationship to Underlying Disease 44
6.3.3.4. Severity of Adverse Events 44
6.3.3.5. Outcome 44
6.3.3.6. Action Taken Regarding the Investigational Product 45
6.3.3.7. Expectedness of an Adverse Event 45
6.3.3.8. Suspected Unexpected Serious Adverse Reactions 45
6.3.4. Recording of Adverse Events 45
6.3.5. Reporting Serious Adverse Events 45
6.3.6. Special Situations 46
6.3.6.1. Overdose 46
6.3.6.2. Adverse Events of Special Interest 46
6.3.6.3. Death 46
6.3.6.4. Unblinding due to a Medical Emergency 47

6.3.7.	Responsibilities of the Investigator	47
6.3.8.	Responsibilities of the Sponsor	47
6.4.	Treatment Overdose	48
6.5.	Pharmacokinetics	48
6.6.	Pharmacodynamics	48
6.7.	[redacted]	48
6.7.1.	[redacted]	49
6.7.2.	[redacted]	49
6.8.	Biomarkers	49
6.9.	[redacted]	49
6.9.1.	[redacted]	49
6.9.2.	[redacted]	50
7.	STATISTICAL CONSIDERATIONS	51
7.1.	Statistical Hypotheses	51
7.2.	Sample Size Determination	51
7.3.	Analysis Populations	52
7.4.	Statistical Analyses	52
7.4.1.	Efficacy Analyses	52
7.4.2.	Safety Analyses	53
7.5.	Interim Analyses	54
7.5.1.	Interim Analysis at Week 12 in Part 1	54
7.5.2.	Interim Analysis at the Completion of Part 1	54
7.5.3.	[redacted]	54
7.5.4.	Interim Analysis Based on Regulatory Interactions	54
7.6.	Data Monitoring Committee	54
8.	DISCONTINUATION OF STUDY INTERVENTION AND PARTICIPANT DISCONTINUATION/WITHDRAWAL	55
8.1.	Discontinuation of Study Intervention	55
8.2.	Participant Discontinuation/Withdrawal from the Study	55
8.3.	Lost to Follow-up	55
9.	SUPPORTING DOCUMENTATION AND OPERATIONAL CONSIDERATIONS	57
9.1.	Appendix 1: Regulatory, Ethical, and Study Oversight Considerations	57

9.1.1. Clinical Monitoring of the Study 57
9.1.2. Regulatory and Ethical Considerations 57
9.1.3. Financial Disclosure 57
9.1.4. Informed Consent Process 58
9.1.5. Data Protection 59
9.1.5.1. Data Safety Monitoring Plan 59
9.2. Committees Structure 59
9.2.1. Data Monitoring Committee 59
9.2.2. Dissemination of Clinical Study Data 59
9.2.2.1. Final Study Report 59
9.2.3. Source Documents 59
9.2.4. Publication Policy 59
9.3. Appendix 2: [redacted] 60
10. REFERENCES 61

LIST OF TABLES

Table 1: Schedule of Activities – Part 1 16
[redacted] [redacted] 18
[redacted] [redacted] 20

LIST OF ABBREVIATIONS AND DEFINITIONS OF TERMS

Abbreviation	Definition
AAV	adeno-associated virus
AE	adverse event
AESI	adverse event of special interest
aHUS	atypical hemolytic uremic syndrome
ALT	alanine aminotransferase
AST	aspartate aminotransferase
CFR	Code of Federal Regulations
CK	creatine kinase
CRF	case report form
CRO	Contract research organization
DMC	data monitoring committee
DMD	Duchenne muscular dystrophy
DNA	deoxyribonucleic acid
ECG	electrocardiogram
ECHO	echocardiogram
GCP	Good Clinical Practices
GGT	gamma-glutamyl transferase
GLDH	glutamate dehydrogenase
HIV	human immunodeficiency virus
ICF	informed consent form
ICH	International Council on Harmonisation
IEC	Independent Ethics Committee

Abbreviation	Definition
IF	immunofluorescence
IP	investigational product
IRB	Institutional Review Board
ITT	intent-to-treat
IV	intravenous(ly)
IXRS	interactive voice/web response system
[redacted]	[redacted]
NOAEL	no observable adverse effect level
NSAA	North Star Ambulatory Assessment
PCS	potentially clinically significant
PDPF	percent dystrophin positive fibers
PI	Principal Investigator
[redacted]	[redacted]
qPCR	quantitative polymerase chain reaction
[redacted]	[redacted]
SAE	serious adverse event
SAP	Statistical Analysis Plan
SoA	Schedule of Activities
SUSARs	suspected unexpected serious adverse reactions
TEAE	treatment-emergent adverse event
ULN	upper limit of normal
WBC	white blood cell

1. PROTOCOL SUMMARY

1.1. Synopsis

Protocol Title: A Multicenter, Randomized, Double-Blind, Placebo-Controlled Trial for Duchenne Muscular Dystrophy Using SRP-9001

Short Title: A Randomized, Double-Blind, Placebo-Controlled Study of SRP-9001 for Duchenne Muscular Dystrophy

Rationale: Based on nonclinical studies in mice and canine species deficient in dystrophin and from the 2006 Duchenne muscular dystrophy (DMD) trial (Mendell 2010) and the ongoing microDys-IV-001 clinical trial, data supports the use of gene transfer for micro-dystrophin expression in skeletal and cardiac muscle.

Objectives and Endpoints: This study will assess the safety and efficacy of exogenous gene transfer in DMD patients by measuring biological and clinical endpoints in 3 parts: two 48-week randomized, double-blinded, placebo-controlled periods (Part 1 and 2), and an open-label follow-up period (Part 3). The primary objectives of this study are the assessments of the safety and efficacy of intravenous (IV) administration of SRP-9001 in DMD patients.

Objectives	Endpoints
**Safety**	
• To evaluate the safety of SRP-9001	• Treatment-emergent adverse events (TEAEs) and serious adverse events (SAEs)<br>• Select clinical laboratory assessments
**Primary**	
• To evaluate micro-dystrophin expression from SRP-9001 at 12 weeks post dosing (Part 1) as measured by Western blot of biopsied muscle tissue	• Change in quantity of micro-dystrophin protein expression from Baseline to Week 12 (Part 1) as measured by Western blot
• To evaluate the effect of SRP-9001 on physical functional assessments as assessed by the North Star Ambulatory Assessment (NSAA) over 48 weeks (Part 1)	• Change in NSAA total score from Baseline to Week 48 (Part 1)

Objectives	Endpoints
**Secondary**	
• To evaluate the effect of SRP-9001 on physical functional assessments over 48 weeks (Part 1)	• Change in time to rise from the floor from Baseline to Week 48 (Part 1)<br>• Change in time to ascend 4 steps from Baseline to Week 48 (Part 1)<br>• Change in time of 10-meter timed test from Baseline to Week 48 (Part 1)<br>• Change in time of 100-meter timed test from Baseline to Week 48 (Part 1)
• To evaluate micro-dystrophin expression from SRP-9001 at 12 weeks (Part 1) as measured by immunofluorescence (IF) fiber intensity of biopsied muscle tissue	• Change in micro-dystrophin expression from Baseline to Week 12 (Part 1) as measured by IF fiber intensity
• To evaluate micro-dystrophin expression from SRP-9001 at 12 weeks (Part 1) as measured by IF percent dystrophin positive fibers (PDPF) of biopsied muscle tissue	• Change in micro-dystrophin expression from Baseline to Week 12 (Part 1) as measured by IF PDPF

[redacted]

[redacted]

[redacted]

1.2. Schema

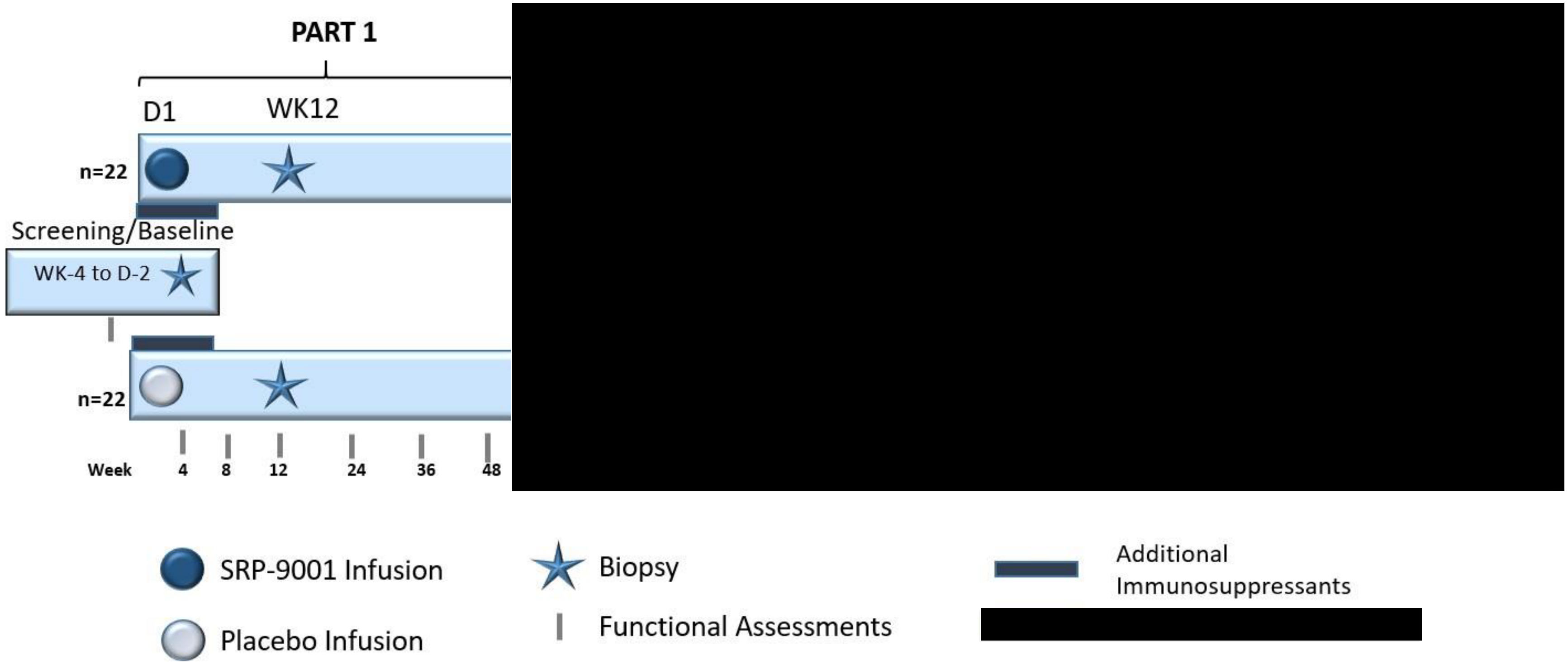

D = day; [redacted]; WK = week.

1.3. Schedule of Activities

Table 1: Schedule of Activities – Part 1

Study Interval	Scr / Baseline	Part 1 - Inpatient			Part 1 - Follow Up											
Study Interval	W-4 to D-2	D-1	D1	D2	W1	W2	W3 [a]	W4	W5 [a]	W6	W8	W10	W12	W24	W36	W48
[redacted]																
Informed consent	X															
Medical history	X															
Vitals	X	X	X	X	X	X		X		X	X	X	X	X	X	X
Height	X	X			X	X		X			X		X	X	X	X
Weight	X	X			X	X		X			X		X	X	X	X
Physical exam	X	X		X	X	X		X		X	X	X	X	X	X	X
[redacted]																
Chest X-ray	X															
Hepatitis B & C, HIV	X															
Clinical lab assessments [b]	X	X		X	X	X		X		X	X	X	X	X	X	X
GLDH [c]	X	X		X	X	X		X		X	X	X	X	X	X	X
Liver function tests [a]							X		X							
Functional assessments [d]	X							X			X		X	X	X	X
[redacted]																
Immunology [e]	X				X	X		X		X	X	X	X	X	X	X
[redacted]																
Muscle biopsy	X												X			
Study treatment infusion			X													
Adverse events	To be collected from time of consent until final study visit.															
Concomitant medications	To be collected from time of consent until final study visit.															

ALT = alanine aminotransferase; AST = aspartate aminotransferase; D/d = day; [redacted] GGT = gamma-glutamyl transferase; GLDH = glutamyl dehydrogenase; HIV = human immunodeficiency virus; [redacted] Scr = screening; W/w = week.

a Liver function tests will be performed at all clinic visits as part of the clinical lab assessments. However, for the Weeks 3 and 5 visits, only liver function tests including GGT, ALT, AST, total bilirubin, albumin, and alkaline phosphate will be performed. The Weeks 3 and 5 visits will be performed either at the clinic or a local laboratory; if collected at the clinic, GLDH should also be collected and processed via a central laboratory .

b Clinical laboratory assessments: Complete blood count/differential/platelet with smear, prothrombin time, partial thromboplastin time, international normalized ratio, electrolytes, ALT, AST, alkaline phosphatase, amylase, lactate dehydrogenase, C-reactive protein, blood urea nitrogen, creatine kinase (preferably on 2-day visits but may be tested on a 1-day visit per Principal Investigator discretion), creatinine, cystatin C, GGT, glucose, total protein, albumin, total bilirubin, urinalysis, complement (CH50, C3, C4, and factor B). Note that CH50 may be collected and analyzed by the clinical site; however, C3, C4, and factor B will be collected and analyz ed by the central laboratory only.

c GLDH assay will be performed by a central laboratory and only collected at clinic visits. If the Weeks 3 and 5 visits occur in the clinic, a blood sample for the assessment of GLDH will be collected.

d Functional Assessments: Time to rise from floor, time to ascend 4 steps, North Star Ambulatory Assessment, 10 -meter timed test, 100-meter timed test.

e Antibodies to rAAVrh74 and antigen-specific T-cells to rAAVrh74 capsid or micro-dystrophin.

[redacted]

Vitals	X	X	X	X	X		X		X	X	X	X	X	X	X
Height	X			X	X		X			X		X	X	X	X
Weight	X			X	X		X			X		X	X	X	X
Physical exam	X		X	X	X		X		X	X	X	X	X	X	X
[redacted]															
Study treatment infusion		X													
[redacted]															
Adverse events	To be collected from time of consent until final study visit.														
Concomitant medications	To be collected from time of consent until final study visit.														

[redacted] D/d = day; [redacted] W/w= week.

[redacted]

[redacted]

[redacted]

Vitals	X	X	X	X	X	X	X	X
Height	X	X	X	X	X	X	X	X
Weight	X	X	X	X	X	X	X	X
Physical exam	X	X	X	X	X	X	X	X

[redacted]

Adverse events	To be collected from time of consent until final study visit.
Concomitant medications	To be collected from time of consent until final study visit.

[redacted] W/w = week.

[redacted]

2. INTRODUCTION

SRP-9001 is a nonreplicating, recombinant adeno-associated virus (AAV) that is being developed for treatment in patients with Duchenne muscular dystrophy (DMD).

2.1. Study Rationale

There is a strong rationale for systemic gene delivery in young DMD boys based on studies of the natural history of this disease. Gene replacement therapy has been studied for the past 10 to 15 years and shows very favorable results in nonclinical studies in mice and canine species deficient in dystrophin.

The goal of SRP-9001 therapy is to increase the expression level of the micro-dystrophin protein in skeletal and cardiac muscle in order to increase strength and protect from contraction-induced injury. Correction of the underlying genetic defect in DMD using gene replacement with SRP-9001 is the most promising treatment. Duchenne muscular dystrophy affects all skeletal muscles in the body, in addition to the diaphragm and heart. As such, a systemic approach is necessary in order to provide the best possible prospect of direct benefit to patients. Utilizing the rAAVrh74 serotype allows for efficient transduction of cardiac, skeletal, and diaphragm muscle without the increased risk of regional delivery strategies. Increased dystrophin expression in vivo may potentially improve patient's muscle function. SRP-9001 appears to have a favorable safety profile and to be generally well-tolerated in nonclinical studies.

All patients will be screened for pre-existing immunity to rAAVrh74 to avoid potential immune reaction and rejection following transfer of the gene.

2.2. Background

Duchenne muscular dystrophy is the most common, severe childhood form of muscular dystrophy. Inheritance follows an X-linked recessive pattern. Birth prevalence has been estimated at 1 in 3500 to 5000 live male births (Mendell 2010). Approximately one-third of cases represent new mutations of the *DMD* gene with the remaining inherited on the X chromosome from a carrier mother. Questions usually begin to surface between 3 to 5 years of age regarding reduced motor skills that alert a need for diagnostic evaluation. Earlier recognition results from delay in motor milestones or family history. At times aminotransaminases are found to be elevated that lead to detection of creatine kinase (CK) elevation and subsequent further testing. Duchenne muscular dystrophy is relentlessly progressive with loss of ambulation by 12 years of age (Brooke 1983). Historically, patients died from respiratory complications. Now, a variety of factors protect the respiratory system related to improved supportive equipment, antibiotics, vaccines, and other ancillary methods (Eagle 2002). Consequently, patients tend to live longer but eventually succumb to cardiac failure (D'Amario 2017).

2.3. Benefit/Risk Assessment

Nonclinical data and results from clinical study SRP-9001-101 suggest a positive benefit/risk relationship and support clinical investigation of SRP-9001 gene replacement in DMD patients.

More detailed information about the known and expected benefits and risks and reasonably expected adverse events (AEs) of SRP-9001 may be found in the Investigator’s Brochure.

3. STUDY DESIGN

3.1. Overall Design

This is a randomized, double-blind, placebo-controlled, 3-part clinical study of systemic gene delivery of SRP-9001 in up to 44 DMD patients 4 to 7 years of age (inclusive) who either have a confirmed frameshift (deletion or duplication) between exons 18 to 58, or premature stop codon mutation between exons 18 to 58. Patients meeting all eligibility criteria will be randomized to SRP-9001 or placebo by the interactive voice/web response system (IXRS). All patients will have the opportunity to receive intravenous (IV) SRP-9001 (1.33×10^{14} vg/kg) in either Part 1 or Part 2. Part 3 of the study will be an open-label follow-up period.

The Sponsor has developed a quantitative polymerase chain reaction (qPCR) method using a linear standard for dose titering. This method replaces the previously used qPCR method which used a supercoiled standard for dose titering developed by Nationwide Children's Hospital. The scale for the linear standard method is different from the previous supercoiled standard method. For patients who received a dose in this study based on Nationwide Children's Hospital qPCR method, the dose used was 2×10^{14} vg/kg which corresponds to 1.33×10^{14} vg/kg as measured by the Sponsor's qPCR method using a linear standard. Note that both numbers (ie, 2×10^{14} and 1.33×10^{14} vg/kg) refer to the same quantity of drug given to the patients; only the effective scale for the 2 methods is different. Hence, as of Amendment 5, Version 6, the reassigned dose for the additional patients in the study is 1.33×10^{14} vg/kg, and for the duration of this study, all references to dosing will use 1.33×10^{14} vg/kg.

3.1.1. Screening/Baseline Period

The Screening/Baseline period will be up to 4 weeks before Day -1 in Part 1. After obtaining informed consent, patients will be evaluated for eligibility. Screening will include collection of demographics and medical history, vital sign measurements, a physical examination, electrocardiogram (ECG), and an echocardiogram (ECHO). Blood and urine samples will be collected for clinical and safety laboratory assessments. Blood samples will also be collected for hepatitis B and C, human immunodeficiency virus (HIV), and antibodies to rAAVrh74 and antigen-specific T-cells to rAAVrh74 capsid and micro-dystrophin. A pretreatment muscle biopsy will involve the gastrocnemius muscle, or a muscle selected by the Principal Investigator (PI) and will be performed after eligibility but before Day -1 in Part 1. The parent/caregiver will be asked to complete questionnaires.

Physical functional assessments will include the time to rise from the floor, time to ascend 4 steps, North Star Ambulatory Assessment (NSAA), the 10-meter timed test, and the 100-meter timed test.

3.1.2. Treatment Period

3.1.2.1. Part 1

Part 1 is a 48-week randomized, double-blind, placebo-controlled period. Patients meeting eligibility requirements will be randomized prior to or on Day -1 to SRP-9001 or placebo in a 1:1 ratio by IXRS. Up to 22 patients will receive IV SRP-9001 (1.33×10^{14} vg/kg) and up to

22 patients will receive a matching volume (up to 10 mL/kg) of placebo (lactated Ringer's solution).

Patients may be admitted to the hospital the day before the study treatment is infused at the discretion of the Investigator. On Day -1, the patient will receive a physical examination, have vital signs collected, and provide blood and urine samples.

The day prior to the study treatment infusion (SRP-9001 or placebo), patient's background dose of steroid for DMD will be increased to at least 1 mg/kg of a glucocorticoid (prednisone equivalent) daily and continue at this level for at least 60 days after the infusion unless earlier tapering is judged by the PI to be in the best interest of the patient.

SRP-9001 or placebo will be administered IV on Day 1 over 1 to 2 hours. Vital signs will be monitored during and after the infusion according to the Study Operations Manual.

On the day after the infusion (Day 2), patients will receive a physical examination, have vital signs collected, and provide blood and urine samples before being discharged or in clinic, as applicable.

Patients will be followed for 48 weeks in Part 1. Patients will complete clinic follow-up visits at the following time points: Weeks 1, 2, 4, 6, 8, 10, 12, 24, 36, and 48 (relative to the infusion on Day 1). In addition, at Weeks 3 and 5, patients will complete follow-up visits for assessment of liver function tests. If the Weeks 3 and 5 visits occur in the clinic, a blood sample for the assessment of glutamate dehydrogenase (GLDH) will be collected.

All patients will have a muscle biopsy performed at Week 12 in Part 1. [redacted]

All patients, parents, caregivers, the Examining Team, the Treating Team, physiotherapists, and clinical staff will be blinded to the treatment the patient receives (SRP-9001 or placebo). The study will be unblinded when the last patient completes the Part 2 Week 48 visit. Refer to the study unblinding plan for additional details.

Safety will be assessed by monitoring of treatment-emergent adverse events (TEAEs), serious adverse events (SAEs), and select laboratory assessments.

Refer to Section 6 Study Assessments and Procedures for additional information.

3.1.2.2. Part 2

Part 2 of the study will begin after the patient completes Part 1. Patients who were randomized to placebo during Part 1 will receive IV SRP-9001 (1.33×10^{14} vg/kg) in Part 2. To maintain blinding throughout the study, patients previously randomized and treated with SRP-9001 in Part 1 will receive up to 10 mL/kg placebo (lactated Ringer's solution). Infusions will be given in the same manner as in Part 1.

[redacted]

If the period between the Week 48 (Part 1) visit and the Day -1 (Part 2) visit is ≤ 4 weeks, only the following assessments are required at the Day -1 (Part 2) visit:

- Vital signs, height, weight
- Physical examination
- [redacted]

[redacted]

Safety will be assessed by monitoring of TEAEs, SAEs, and select laboratory assessments.

Refer to Section 6 Study Assessments and Procedures for additional information.

3.1.2.3. Part 3

Part 3 of the study will be an open-label follow-up period. The study will be unblinded once the last patient completes the Part 2 Week 48 visit; refer to the study unblinding plan for additional

details. [redacted]

3.2. Scientific Rationale for Study Design

Based on the hypothesis that micro-dystrophin expression can improve sarcolemmal integrity resulting in improved cardiac and skeletal muscle function as well as preliminary results from the ongoing study (Study SRP-9001-101), this study will further assess the safety and efficacy of exogenous gene transfer in DMD patients measuring biologic and clinical efficacy endpoints.

3.3. [redacted]

[redacted]

[redacted]

[redacted]

[redacted]

3.4. End of Study Definition

The end of the study is defined as the date of last patient, last visit. A patient's last visit is dependent upon when that patient received SRP-9001 infusion:

- For patients who receive SRP-9001 during Part 1, the last visit will be the Week 212 visit in Part 3.
- For patients who receive SRP-9001 during Part 2, the last visit will be the Week 260 visit in Part 3.

4. STUDY POPULATION

Prospective approval of protocol deviations to recruitment and enrollment criteria, also known as protocol waivers or exemptions, are not permitted.

4.1. Inclusion Criteria

Participants are eligible to be included in the study only if all of the following criteria apply:

1. Male, 4 to 7 years of age, inclusive, at the time of screening in Part 1.
2. Molecular characterization of the DMD gene with either frameshift (deletion or duplication) between exons 18 to 58, or premature stop codon mutation between exons 18 to 58.
3. Indication of symptomatic muscular dystrophy:
   - [redacted]
   - [redacted]
4. Ability to cooperate with motor assessment testing.
5. Stable dose equivalent of oral corticosteroids for at least 12 weeks before screening and the dose is expected to remain constant (except for potential modifications to accommodate changes in weight) throughout Part 1 and 2 of the study.

4.2. Exclusion Criteria

Participants are excluded from the study if any of the following criteria apply:

1. Signs of cardiomyopathy, including ECHO [redacted]
2. [redacted]
3. [redacted]
4. [redacted]
5. Has a medical condition or extenuating circumstance that, in the opinion of the Investigator, might compromise the patient's ability to comply with the protocol required testing or procedures or compromise the patient's wellbeing, safety, or clinical interpretability.
6. [redacted]
7. [redacted].
8. Has received any investigational medication (other than corticosteroids) or exon skipping medications (including EXONDYS 51®), experimental or otherwise, within 6 months of screening.
9. Has received any type of gene therapy, cell-based therapy (eg, stem cell transplantation), or CRISPR/Cas9 therapy.

10. Abnormal laboratory values considered clinically significant:
    - [redacted]
    - [redacted]
    - [redacted]
    - [redacted]
    - [redacted]
    - [redacted]
11. [redacted]
12. [redacted].

4.3. Lifestyle Considerations

Serum blood draws will be taken to measure CK levels throughout the study. Every effort should be made to collect these samples on the first day of a 2-day study visit; however, per PI discretion, the samples may be collected during a 1-day study visit. Creatine kinase samples must be collected before any functional assessments are performed. Parents or caregivers should be asked to limit patient's physical activity level over the 3 days before a visit where CK samples will be collected.

4.4. Screen Failures

Screen failures are defined as participants who consent to participate in the clinical study but are not subsequently randomly assigned to study intervention.

Individuals who do not meet the eligibility criteria may be rescreened at the Investigator's discretion.

5. STUDY INTERVENTION

Study intervention is defined as any investigational intervention(s), glucocorticoid(s), or placebo intended to be administered to a study participant according to the study protocol.

5.1. Study Intervention(s) Administered

	Micro-dystrophin	Placebo	Corticosteroid
**Intervention Name**	SRP-9001 gene vector	Lactated Ringer's solution	Glucocorticoid
**Type**	Vector	Placebo	Immunosuppressant
**Dose Formulation**	IV infusion	IV infusion	Oral or IV
**Dose** [a]	1.33×10^{14} vg/kg	N/A	1 mg/kg (prednisone equivalent)
**Dosage Level(s)**	Single dose	Single dose	Day before infusion and continuing for at least 60 days after the infusion unless earlier tapering is judged by the Principal Investigator to be in the best interest of the patient
**Route of Administration**	IV infusion (peripheral limb vein)	IV infusion (peripheral limb vein)	Oral or IV

IV = intravenous; N/A = not applicable; qPCR = quantitative polymerase chain reaction.

a For patients who received a dose in this study based on Nationwide Children's Hospital qPCR method, the dose used was 2×10^{14} vg/kg which corresponds to 1.33×10^{14} vg/kg as measured by the Sponsor's qPCR method using a linear standard.

5.1.1. Pre-infusion Immunosuppressants

The day before the infusion, the patient's background dose for DMD will be increased to at least 1 mg/kg of a glucocorticoid (prednisone equivalent).

5.1.2. Post-infusion Immunosuppressants

Patients will remain on their stable dose of corticosteroids throughout Part 1 and 2 of the study but will be increased to at least 1 mg/kg of a glucocorticoid (prednisone equivalent) for at least 60 days after the infusion unless earlier tapering is judged by the PI to be in the best interest of the patient. Additional glucocorticoid may be utilized if GGT level is > 150 U/L or there are other clinically significant liver function abnormalities following infusion. A tapering dose of glucocorticoid will be implemented based on individual patients' immune response to the infusion, assessed by liver function monitoring with GGT. This monitoring will be done by the Treating Team who will also monitor AEs. If there are signs of immune response in specific

organ systems like the liver, the Treating Team will closely monitor and make adjustments in glucocorticoid dosing.

5.1.3. Intravenous Infusion (Day 1, Part 1 and Part 2)

SRP-9001 or placebo administration will be through a peripheral limb vein. Administration will be performed according to the Site-Specific Pharmacy and/or Dose Administration Manual.

5.1.4. Post Infusion Monitoring

5.1.4.1. Immediately Following Infusion

Vital signs will be monitored during and after the infusion according to the Study Operations Manual. Concomitant medications and all AEs and SAEs will also be monitored and documented following injection.

5.1.4.2. [redacted]

[redacted]

5.2. Preparation/Handling/Storage/Accountability

Compliance standards for Biosafety Level 1 vectors must be followed according to standard pharmacy protocol for handling investigational products similar to SRP-9001.

5.3. Measures to Minimize Bias: Randomization and Blinding

5.3.1. Establish Patient Identification Number

All patients will be given a unique sequentially assigned patient identifier. Patients will be identified only by this number in order to protect identity.

5.3.2. Assignment to a Treatment Group

This is a double-blind study. Patients who have signed the informed consent and who meet all eligibility will be randomized using the IXRS to 1 of the 2 treatment groups prior to or on Day -1 in Part 1.

5.3.3. Randomization

Patients will be randomized in a 1:1 ratio by the IXRS in Part 1 of the study to receive either SRP-9001 or placebo. Patients randomized to SRP-9001 in Part 1 of the study will receive placebo in Part 2. Patients randomized to placebo in Part 1 of the study will have the opportunity to receive SRP-9001 in Part 2.

Randomization will be stratified by age group at Baseline (4-5 vs 6-7 years). To ensure approximately equal numbers of patients are assigned to the 2 treatment groups (SRP-9001 vs placebo) in Part 1, each age group must have a multiple of 4 patients.

5.3.4. Blinding/Unblinding

All patients and study staff will be blinded to treatment assignments with the exception of the pharmacist until the last patient completes the Part 2 Week 48 visit. The blind of the treatment may be broken only in exceptional circumstances, such as when knowledge of the study treatment is essential for treating a patient due to an SAE. If time permits, the Investigator must contact the unblinded CRO Medical Director before unblinding of the patient. If time does not permit, the Investigator may authorize breaking of the blind and then notify the unblinded CRO Medical Director. The study treatment information should be disclosed only to personnel who need the information for the medical care of the patient. If the study treatment is unblinded, the patient number, time, date, and reason for unblinding must be recorded in the study records. Study personnel who were unblinded should be identified. Refer to the study unblinding plan for further details regarding the unblinding of the study for Part 3.

Separate study personnel will be designated to conduct functional assessments and to treat patients in order to protect against possible unblinding of treatment assignment during regular clinical care of patients.

The PI will designate the following investigational site personnel:

- An Examining Team
- A Treating Team

The Examining Team will be:

- Composed of physiotherapists trained in the proper execution of the physical functional assessments (NSAA, 10-meter timed test, 100-meter timed test, time to rise from floor, and time to ascend 4 steps)

The Treating Team will be:

- Composed of clinicians who will be responsible for the following:
    - Administering the study treatment
    - Conducting the patient's follow-up visits but not the physical functional assessments at those visits per the SoA (Section 1.3)
    - Review of all clinical and safety laboratory results
    - Glucocorticoid dose adjustments
    - Assessment of AEs, SAEs, and TEAEs

- Management of the routine care of the patient

The physiotherapists from the Examining Team will be responsible for conducting the physical functional assessments (NSAA, 10-meter timed test, 100-meter timed test, time to rise from floor, and time to ascend 4 steps). They will not have access to any patient data and must be restricted from having access to AEs, concomitant medications, laboratory data, and any other data that have the potential of revealing the treatment assignment. The physiotherapists should be instructed not to have conversations with the patients and parents/caregivers about their progress in the study.

The Treating Team members will be responsible for the clinical care of the patient. They will manage the study drug infusion, steroid dosing changes, review all laboratory data, assess AEs and SAEs, and perform all assessments except for the physical functional assessments (NSAA, 10-meter timed test, 100-meter timed test, time to rise from floor, and time to ascend 4 steps). The Treating Team will not have access to the data collected by the Examining Team.

The study will be unblinded once the last patient completes the Part 2 Week 48 visit; refer to the study unblinding plan for further details.

5.4. Concomitant Therapy

To be collected from the time of consent until final study visit, recorded on the case report form (CRF).

Any medication or vaccine (including over the counter or prescription medicines, vitamins, and/or herbal supplements) that the participant is receiving at the time of enrollment or receives during the study must be recorded in the CRF.

If a reaction to the infusion occurs, the Treating Team should treat the reaction. The date and time of medication administration as well as the name and dosage regimen must be recorded.

5.4.1. Rescue Medicine

There are no specific rescue medications for use in this study that will turn off SRP-9001 transgene expression. Corticosteroid therapy, at the PI's discretion, may be used to manage possible liver-associated adverse effects of SRP-9001.

5.5. Dose Modification

5.5.1. Stopping Rules

Study enrollment will be interrupted when any patient experiences 1 or more Grade 3 (severe) or higher AEs that are unexpected as related to gene therapy or DMD. This will include any patient death, important clinical laboratory findings, or any severe local complication in the injected area related to administration of the study agent. The findings will be reported to the data monitoring committee (DMC). If after review by the DMC, the recommendation is made to continue, the Sponsor may decide that the study will proceed.

5.5.2. Dosing Schedule

Based on the safety profile of systemic gene delivery clinical trial (Study SRP-9001-101) for DMD using SRP-9001, there will be at least a 1-week interval between dosing each patient for the first 4 patients.

5.6. Intervention after the End of the Study

There will be no intervention following the end of the study.

6. STUDY ASSESSMENTS AND PROCEDURES

- Study procedures and their timing are summarized in the SoA (Section 1.3). Protocol waivers or exemptions are not allowed.
- Immediate safety concerns should be discussed with the Sponsor immediately upon occurrence or awareness to determine if the participant should continue or discontinue study intervention.
- Adherence to the study design requirements, including those specified in the SoA, is essential and required for study conduct.
- All screening evaluations must be completed and reviewed to confirm that potential participants meet all eligibility criteria. The Investigator will maintain a screening log to record details of all participants screened and to confirm eligibility or record reasons for screening failure, as applicable.
- Procedures conducted as part of the participant's routine clinical management (eg, blood count) and obtained before signing of the informed consent form (ICF) may be utilized for screening or baseline purposes provided the procedures met the protocol-specified criteria and were performed within the time frame defined in the SoA.

6.1. Efficacy Assessments

Planned time points for all efficacy assessments are provided in the SoA (Section 1.3).

6.1.1. Muscle Biopsy

A muscle biopsy will be performed at Screening/Baseline after establishing eligibility. Muscle biopsies will be used to quantify transgene expression by Western blot comparing the baseline biopsy with the biopsies collected at Week 12 in Part 1 [redacted] Additional information can be found in the Biopsy Manual.

6.1.2. Immunofluorescence Fiber Intensity / Immunofluorescence PDPF

Muscle biopsies will be used to quantify transgene expression by immunofluorescence (IF) fiber intensity and IF percent dystrophin positive fibers comparing the baseline biopsy with both the biopsies collected at Week 12 in Part 1 [redacted]

[redacted]

[redacted]

[redacted]

[redacted]

6.1.5. Physical Functional Assessments

Efficacy will be measured by the following functional tests: time to rise from the floor, ascend 4 steps, NSAA, 10-meter timed test, and 100-meter timed test. Assessments will be administered by a physiotherapist. The physiotherapist will not have access to information that can disclose study treatment, laboratory results, or adverse events.

The patient's functional assessments will be videotaped for quality control and may be viewed by government agencies if the study goes through a quality review. These videos may also be used to demonstrate any functional outcomes related to the study drug and shared at scientific meetings and/or with regulatory authorities.

6.1.5.1. Time to Rise from the Floor

This assessment is performed as a part of the NSAA. It measures the time taken to rise from supine to standing.

6.1.5.2. Ascend 4 Steps

This assessment measures the time taken to climb 4 steps.

6.1.5.3. North Star Ambulatory Assessment

The NSAA is a healthcare provider administered scale that rates patient performance on various functional activities (Mazzone 2010). During this assessment, patients will be asked to perform 17 different functional activities, including a 10-meter walk/run, rising from a sitting position to standing position, standing on 1 leg, climbing a box step, descending a box step, rising from lying to sitting, rising from the floor, while supine lifting head off floor, standing on heels, and jumping.

6.1.5.4. 10-meter Timed Test

This assessment measures the time needed to move 10 meters.

6.1.5.5. 100-meter Timed Test

This assessment measures the time needed to move 100 meters.

6.2. Safety Assessments

Planned time points for all safety assessments are provided in the SoA (Section 1.3).

Safety parameters evaluated during the conduct of the study include AEs, SAEs, TEAEs, clinical laboratory values, antibody titers, vital signs, ECGs, ECHOs, physical examination findings, and use of concomitant medications.

6.2.1. Physical Examinations

Physical examinations will be conducted at the time points specified in the SoA (Section 1.3). Physical examinations will include examination of general appearance, head, ears, eyes, nose, and throat, heart, chest, abdomen, skin, lymph nodes, extremities, musculoskeletal, and neurological systems.

Any physical examination finding assessed as clinically significant should be recorded as an AE or SAE. If SAE criteria are met, the event should be recorded and reported according to the SAE reporting process (see Section 6.3).

6.2.2. Vital Signs

Vital signs (blood pressure, heart rate, respiration, and oral temperature), will be measured at the time points specified in the SoA (Section 1.3).

Vital signs including blood pressure, heart rate, respiratory rate, and temperature will be performed after study treatment infusion according to the Study Operations Manual.

Any vital signs assessed as clinically significant should be recorded as an AE or SAE. If SAE criteria are met, the event should be recorded and reported according to the SAE reporting process (see Section 6.3).

6.2.3. Electrocardiograms and Echocardiograms

Single ECGs will be obtained at the time points specified in the SoA (Section 1.3) using an ECG machine that automatically calculates the heart rate and measures PR, QRS, and QT intervals. ECGs will be performed at a consistent time of day throughout the study. The Investigator, or designee, will review the results of the ECG report and determine if the findings are clinically significant.

A standard 2-dimensional ECHO will be obtained at the time points specified in the SoA (Section 1.3). Echocardiograms will be performed at a consistent time of day throughout the study and before any invasive procedures (eg, blood sampling, study drug infusion, or biopsy). The ECHO will be reviewed and interpreted by local medically qualified personnel. Left ventricular ejection fraction will be noted. The Investigator, or designee, will review the results of the ECHO report and determine if the findings are clinically significant.

Clinical significance is defined as any variation in assessment results that has medical relevance resulting in an alteration in medical care. If clinically significant deterioration from baseline levels is noted, the Investigator will continue to monitor the patient with additional assessments until:

- Values have reached normal range and/or baseline levels; or
- In the judgment of the Investigator together with the Sponsor's Medical Monitor, abnormal values are assessed to be not related to the administration of study treatment or other protocol-specific procedures, and additional assessments are not medically indicated. In the judgement of the Investigator, an unexplained laboratory abnormality that could potentially lead to a poor outcome should result in interruption of treatment.

If assessed as clinically significant, the event should be recorded as an AE or SAE as required in Section 6.3.

6.2.4. Clinical Safety Laboratory Assessments

The following routine clinical laboratory tests will be performed at the time points specified in the SoA (Section 1.3). Samples will be collected and processed according to the local laboratory

procedures, the Study Operations Manual, and/or the Central Laboratory Manual provided for the study:

Chemistry:	Sodium, chloride, potassium, carbon dioxide, glucose, creatinine, blood urea nitrogen, total protein, albumin, total bilirubin, alkaline phosphatase, amylase, ALT, AST, GGT, lactate dehydrogenase, C-reactive protein, CK, and serum cystatin C, complement (CH50, C3, C4, and factor B), and GLDH<br>Note: CH50 may be collected and analyzed by the clinical site; however, C3, C4, and factor B will be collected and analyzed by the central laboratory only.<br>Note: The GLDH assay will be performed by a central laboratory and only at clinic visits.
Hematology:	Red blood cells, total WBCs, hemoglobin, hematocrit, neutrophils, lymphocytes, monocytes, eosinophils, basophils, platelets, and abnormal cells
Coagulation Screen:	Prothrombin time, partial thromboplastin time, and international normalized ratio
Immunology	Antibodies to rAAVrh74; antigen-specific T-cells to rAAVrh74 capsid and micro-dystrophin
Urinalysis:	pH, specific gravity, protein, glucose, ketones, cytology, and hemoglobin

Any laboratory abnormality deemed clinically significant by the Investigator should be recorded as an AE. A clinically significant abnormality is an abnormality confirmed by repeat testing, that in the judgment of the Investigator warrants a change in management. This alteration may include monitoring the laboratory test further, initiating other diagnostic tests or procedures, changing ongoing treatment, or administering new treatment(s).

Whenever possible, the underlying medical diagnosis (eg, anemia) should be recorded as the AE term. Repeated additional tests and/or other evaluations required to establish the significance and etiology of an abnormal result should be obtained when clinically indicated.

Patients enrolled under this clinical protocol are expected to present clinically with adverse events related to natural progression of the disease. The draft guidance entitled “Duchenne Muscular Dystrophy Developing Drugs for Treatment over the Spectrum of Disease” (https://www.regulations.gov/document?D=FDA-2014-D-1264-0002) provides the basis of expected disease-related AEs. The expectedness of all AEs will be determined according to the most recent versions of the Investigator’s Brochure (Section 6.3.3.7).

Asymptomatic elevations in transaminases are a feature of Duchenne muscular dystrophy, as they are related to release of AST and ALT from muscle tissue and correlate with levels of CK. These are a feature of the disease itself. Levels of AST and ALT up to 10× the ULN are expected to be seen at Baseline and are not indicative of muscle or liver injury. Elevated AST/ALT levels are thus not exclusionary for enrollment and, depending on circumstances, will not be recorded as adverse events during the course of the trial.

6.2.5. Safety Monitoring

6.2.5.1. Safety Monitoring for Liver Chemistry Tests

Liver chemistry tests need to be monitored as specified in the SoA (Section 1.3). Initial abnormal liver chemistry test result(s) need to be confirmed if:

- GGT or GLDH is > 3 × ULN at any time during the study
- AST or ALT measurement is > 2 × baseline value if the baseline value is >ULN

Patients with confirmed liver chemistry test results (as above) should have their liver chemistry tests (GGT, GLDH, ALT, AST, alkaline phosphatase, international normalized ratio or prothrombin time, and total and direct bilirubin) retested at a minimum of 2 times weekly and additional testing beyond that is at the discretion of the Investigator. Frequency of retesting can decrease to once a week or less if abnormalities stabilize and the patient is asymptomatic.

Additional Investigations

Patients with confirmed abnormal liver chemistry test results (as above) are recommended to have the following evaluations performed:

- Obtaining a more detailed history of symptoms and prior or concurrent diseases
- Obtaining a history of concomitant drug use (including nonprescription medications and herbal and dietary supplement preparations), alcohol use, recreational drug use, and special diets
- Ruling out acute viral hepatitis types A, B, C, D, and E; autoimmune hepatitis; Non-Alcoholic Steato Hepatitis; hypoxic/ischemic hepatopathy; and biliary tract disease
- Consideration of other viral illnesses that have been associated with hepatitis (eg, Epstein-Barr virus, cytomegalovirus, Human Herpesvirus 6)
- Obtaining a history of exposure to environmental chemical agents

Additional liver evaluations, including gastroenterology/hepatology consultations, hepatic computed tomography or [redacted] scans, may be performed at the discretion of the Investigator, in consultation with the Sponsor Medical Monitor.

Refer to Section 6.3.6.2 for the list of adverse events of special interest (AESIs) related to liver chemistry tests.

6.2.5.2. Safety Monitoring for Hypersensitivity and Complement-Mediated Reactions

Patients will be monitored for hypersensitivity and complement-mediated reactions as follows:

- Hypersensitivity: Patients will be monitored for occurrence of allergic reactions by monitoring AEs as specified in the SoA (Section 1.3). Patients will be instructed to promptly report any signs or symptoms of fever or constitutional symptoms that may arise during the study, and the Investigator needs to closely evaluate all potential causes, including concomitant illness.

- Complement-mediated reactions: Patients will be monitored for occurrence of complement-mediated reactions by monitoring AEs as specified in the SoA (Section 1.3). In addition to monitoring AEs, routine laboratory monitoring for complement levels (CH50, C3, C4, and factor B) will be performed as outlined in the SoA (Section 1.3).

Additional Investigations

- Hypersensitivity: Patients who experience significant or persistent constitutional symptoms, including any Grade ≥ 3 hypersensitivity reactions, need to be discussed with the Sponsor Medical Monitor to determine whether additional monitoring or laboratory tests are required. Additional evaluations including immunology consultation, tests for allergic reactions (eg, absolute eosinophils, eosinophilic cationic protein, serum/plasma tryptase) may be performed at the discretion of the Investigator in consultation with the Sponsor Medical Monitor.
- Atypical hemolytic uremic syndrome (aHUS): Patients diagnosed with aHUS need to be discussed with the Sponsor Medical Monitor to determine additional monitoring and laboratory testing. Additional evaluations including nephrology consultation and tests for complement activation and alternative etiology (C3a, C5a, Bb, prothrombin time, activated partial thromboplastin time, fibrin degradation products, D-dimer, ADAMTS13, complement factor H Ab, shiga toxin-producing *Escherichia coli*, stool culture swab, antiphospholipid antibody, ECG, ECHO, antinuclear antibodies, anticentromere antibodies, anti-scl-70, anti-ds-DNA, plasma homocysteine, plasma and urine methylmalonic acid, G6PD activity, T antigen, testing for influenza, cytomegalovirus, and/or Epstein-Barr virus) may be performed at the discretion of the Investigator in consultation with the Sponsor Medical Monitor.

Refer to Section 6.3.6.2 for the list of AESIs related to hypersensitivity.

6.2.5.3. Safety Monitoring for Platelet Count Results

Platelet counts will be monitored according to the SoA (Section 1.3). Patients with a confirmed occurrence of platelets < 75, 000/mm^3 need to have the following evaluations performed:

- Complete blood count with reticulocytes
- Peripheral blood smear
- Coagulation panel (prothrombin time/international normalized ratio, activated partial thromboplastin time)
- High-sensitivity C-reactive protein

Additional Investigations

Additional platelet evaluations for confirmed, unexplained significant platelet count reductions, including hematology consultation, fibrinogen, fibrinogen split products/D-dimer, von Willebrand factor, total immunoglobulins, complement levels, viral serologies, auto-antibody screen, antiplatelet antibodies, antiplatelet factor 4 assay, and platelet function tests may be performed at the discretion of the Investigator in consultation with the Sponsor Medical Monitor.

Refer to Section 6.3.6.2 for the list of AESIs related to thrombocytopenia.

6.2.5.4. Safety Monitoring for Rhabdomyolysis

Rhabdomyolysis will be monitored by urine dipstick and assessments of AEs per the time points in the SoA (Section 1.3). Patients who have confirmed heme+ dipstick urinalysis need to be evaluated by urine microscopy and for the following AEs:

- Rhabdomyolysis
- Myoglobinuria
- Chromaturia
- Acute onset or exacerbation of myalgia

Additional Investigations

In case of rhabdomyolysis, myoglobinuria, or chromaturia, patients will need to have evaluations of myoglobinuria, CK, renal function (eg, serum cystatin C), and serum chemistry 2 or 3 times weekly until values reach usual/pre-event levels or stabilize. In case of acute onset or exacerbation of myalgia in the absence of rhabdomyolysis, myoglobinuria, or chromaturia, these evaluations should be undertaken at the discretion of the Investigator.

In addition, Investigators should obtain a more detailed history of symptoms, preceding activity and hydration status, concomitant drug use, and recent or concurrent infections. Additional evaluations, including rheumatology/immunology consultations, and anti-muscle antibodies, may be performed at the discretion of the Investigator in consultation with the Sponsor Medical Monitor.

Refer to Section 6.3.6.2 for the list of AESIs related to rhabdomyolysis.

6.2.6. Immunogenicity Assessment

Immunology studies for post gene transfer antibodies to rAAVrh74 will be performed.

Serum samples will be screened for antibodies binding to rAAVrh74 and the titer of confirmed positive samples will be reported.

The detection and characterization of antibodies to rAAVrh74 will be performed pre and post infusion in Part 1 and Part 2 using a validated assay method. Antibodies may be further characterized and/or evaluated for their ability to neutralize the activity of the study intervention(s). Samples may be stored for a maximum of 15 years (or according to local regulations) following the last participant's last visit for the study at a facility selected by the Sponsor to enable further analysis of immune response to SRP-9001.

Antigen-specific T-cells will be evaluated in peripheral blood mononuclear cells in samples collected from all participants according to the SoA (Section 1.3). An enzyme-linked immune absorbent spot assay to detect and characterize antigen-specific T-cells to rAAVrh74 capsid and micro-dystrophin will be performed using a validated assay method.

[redacted]

[redacted]

[redacted]

[redacted]

[redacted].

6.3. Adverse Events and Serious Adverse Events

6.3.1. Collection of Adverse Events

Over the entire duration of the study, site personnel will ensure that all AEs are recorded appropriately. If an AE occurs, the primary concern is for patient safety, and the Investigator will use his/her judgment and expertise to determine the appropriate course of action.

All AEs from the time of informed consent through the last follow-up visit will be recorded in each enrolled patient's CRF. For consented patients who are found to be ineligible for the study during the Screening period and are not enrolled (ie, Screening failures), only SAEs will be collected.

If, at any time after the patient has completed participation in the study, the Investigator or study staff becomes aware of an SAE that the Investigator believes is possibly/probably or definitely related to the investigational product (IP) or is possibly/probably or definitely related to a study procedure, then the event and any known details must be reported promptly to the Sponsor.

6.3.2. Definition of Adverse Events

6.3.2.1. Adverse Event

An AE is any untoward medical occurrence in a clinical trial participant, which does not necessarily have a causal relationship with the investigational drug. An AE can, therefore, be any unfavorable and unintended symptom, sign, disease, condition, or test abnormality that occurs during or after administration of an IP whether or not considered related to the IP.

Adverse events include:

- Symptoms described by the patient or signs observed by the Investigator or medical staff.
- Test abnormalities (laboratory tests, ECG, X-rays, etc.) that result in an alteration in medical care (diagnostic or therapeutic).

Abnormalities present at Screening are considered AEs only if they reoccur after resolution or worsen during the AE collection period.

6.3.2.2. Serious Adverse Events

An SAE is defined as any AE that results in any of the following:

- **Death**: The patient died as the result of the event.
- **Life-threatening event**: Any AE that places the patient, in the view of the Investigator or Sponsor, at immediate risk of death from the AE as it occurred, ie, does not include an AE that had it occurred in a more severe form, might have caused death.
- **Required or prolonged inpatient hospitalization**: The AE resulted in hospitalization or prolonged an existing hospitalization. Since hospitalization may be part of the study, only hospitalizations that are longer than expected per protocol procedures, based on Investigator judgment, will be considered prolonged hospitalizations.
- **Persistent or significant disability/incapacity**: An AE that results in persistent or significant disability or disruption of a person’s ability to conduct normal life functions.
- **Important medical events**: An AE that may not result in death, be life-threatening, or require hospitalization may be considered an SAE when, based upon appropriate medical judgment, the event may jeopardize the patient and may require medical or surgical intervention to prevent one of the outcomes listed above.

6.3.2.3. Adverse Events of Special Interest

An AESI is any AE (serious or nonserious) that is of scientific and medical interest specific to the study treatment for which ongoing and/or rapid communication by the Investigator to the Sponsor is appropriate. See Section 6.3.6.2 for the list of AESIs for this study.

6.3.3. Classification of Adverse Events

Each AE whether serious or non-serious will be classified by the Investigator according to the following rules and definitions.

6.3.3.1. Relationship to Investigational Product

For each AE, the Investigator will determine whether there is a reasonable likelihood that the AE may have been caused by the study treatment according to the categories below:

**Unrelated:**	The event is clearly not related to the study treatment.
**Possibly/probably related:**	The event could be related/is likely to be related to the study treatment.
**Definitely related:**	The event is clearly related to the study treatment.

6.3.3.2. Relationship to Study Procedures

For each AE the Investigator will determine whether there is a reasonable possibility that the AE may have been caused by the study procedures according to the categories below:

**Unrelated:**	The event is clearly not related to the study procedures.
**Possibly/probably related:**	The event could be related/is likely to be related to study procedures.
**Definitely related:**	The event is clearly related to the study procedures.

6.3.3.3. Relationship to Underlying Disease

For each AE the Investigator will determine whether there is a reasonable possibility that the AE may be related to the underlying disease according to the categories below:

**Unrelated:**	The event is clearly not related to the underlying disease
**Possibly/probably related:**	The event could be related/is likely to be related to the underlying disease
**Definitely related:**	The event is clearly related to the underlying disease

Events of disease progression may be considered AEs, based on the Investigator's discretion.

6.3.3.4. Severity of Adverse Events

Note that severity is not the same as "seriousness".

The Investigator will assess the severity of all AEs as Mild, Moderate, or Severe, based on the following definitions:

**Mild:**	The event does not interfere with the patient's usual activities.
**Moderate:**	The event interferes with the patient's usual activities.
**Severe:**	The event prevents the patient from undertaking their usual activities and requires therapeutic intervention or cessation of the study treatment.

6.3.3.5. Outcome

An outcome describes the status of the AE. The Investigator will provide information regarding the patient outcome of each AE. Outcome categories will include recovered, recovered with sequelae, not recovered, fatal, and unknown.

6.3.3.6. Action Taken Regarding the Investigational Product

The Investigator will provide information regarding the action taken with respect to the study treatment in response to the AE. Categories for action taken regarding study treatment will include none, drug interrupted, drug withdrawn, and not applicable.

6.3.3.7. Expectedness of an Adverse Event

The expectedness of all AEs will be determined according to the most recent versions of the Investigator's Brochure.

6.3.3.8. Suspected Unexpected Serious Adverse Reactions

Suspected unexpected serious adverse reactions (SUSARs) will be reported within the required timelines in an unblinded fashion to regulatory authorities and Institutional Review Board (IRB)/Independent Ethics Committee (IEC) per the requirements of the concerned competent authorities. SUSARs will also be reported in a blinded fashion to study Investigators.

6.3.4. Recording of Adverse Events

All AEs from the time of informed consent/assent through the last follow-up visit will be recorded in each enrolled patient's CRF. For patients who are found to be ineligible for the study during the Screening period and are not enrolled (ie, Screening failures), only SAEs will be recorded.

Information should include: a concise description of the event; date of event onset and resolution; determination of seriousness, severity, corrective treatment (if any), outcome, and relationship to IP or study procedure or underlying disease; and any action taken will be recorded. Resolution occurs when the patient has returned to his baseline state of health or further improvement or worsening of the event is not expected.

Whenever possible, a diagnosis will be recorded as an AE, rather than symptoms or isolated laboratory abnormalities related to that diagnosis. Several symptoms or laboratory results that are related to the same diagnosis can thus be part of the same AE. A medical or surgical procedure is not an AE; rather the condition leading to the procedure should be recorded as the AE. Similarly, death is not an AE, but is rather the outcome of the AE(s) that resulted in death. If the AE(s) leading to death are not known, then death must be reported as an AE. All AEs will be followed until the resolution of AE, completion of the patient's study participation, or study termination, whichever occurs first. SAEs will be followed until resolution or until the condition stabilizes or returns to baseline status.

6.3.5. Reporting Serious Adverse Events

It is the responsibility of the Investigator that reporting is done adequately. In order to meet Regulatory reporting timelines, the study site is obligated to report any SAE(s) to the Sponsor or designee immediately and no later than 24 hours after receiving information of an event that meets at least one of the criteria for seriousness.

6.3.6. Special Situations

6.3.6.1. Overdose

Refer to Section 6.4 for the definition of treatment overdose. An overdose is not an AE. An overdose will be reported even if it does not result in an AE. An overdose will be recorded on the Clinical Trial Safety Reporting Form and sent to the Sponsor or designee within 24 hours.

6.3.6.2. Adverse Events of Special Interest

All AESIs need to be reported to the Sponsor via email to [redacted] within 24 hours of awareness, irrespective of the seriousness of the AE.

In this study, the following AEs are considered of special interest:

Hepatotoxicity

- GGT or GLDH > 8 × ULN
- GGT or GLDH > 5 × ULN and persists for ≥ 2 weeks
- GGT or GLDH > 3 × ULN, and either total bilirubin >2 × ULN or international normalized ratio > 1.5
- GGT or GLDH > 3 × ULN and the new appearance (ie, onset coincides with the changes in hepatic enzymes) of fatigue, nausea, vomiting, right upper quadrant pain or tenderness, fever, rash, or eosinophilia (> 5%) felt by the Investigator to be potentially related to hepatic inflammation

Hypersensitivity

- Anaphylaxis, anaphylactoid reaction, or angioedema
- Any severe (Grade ≥ 3) allergic reaction
- Any severe (Grade ≥ 3) inflammation event (eg, aHUS, acute kidney injury, arteritis, myocarditis, pneumonitis, etc.)

Thrombocytopenia

- Platelet count < 75,000/mm^3

Rhabdomyolysis

- Rhabdomyolysis
- Myoglobinuria
- Chromaturia
- Acute onset or exacerbation of myalgia

6.3.6.3. Death

Death is an outcome of an event. All causes of death are SAEs. In the event of death, every effort should be made to obtain a death certificate and if possible, an autopsy report. If the cause of death is unknown, death will be recorded as the event.

6.3.6.4. Unblinding due to a Medical Emergency

In the event of a medical emergency, such as when knowledge of the patient's treatment assignment is essential for treating a patient due to an SAE, the Investigator has the option to unblind treatment assignment through the IXRS. If time permits, the Investigator must contact the unblinded CRO Medical Director before the unblinding of the patient. If time does not permit, the Investigator may authorize breaking of the blind, and then notify the unblinded CRO Medical Director.

The reasons for unblinding and the study personnel who were unblinded must be noted in the source documentation. The Investigator must not disclose information about treatment assignment to anyone who does not need the information for patient care. Disposition of patients who become unblinded due to medical emergency will be determined following discussion with the Sponsor.

The study will be unblinded once the last patient completes the Part 2 Week 48 visit; refer to the study unblinding plan for further details.

6.3.7. Responsibilities of the Investigator

The responsibilities of the Investigator include but are not limited to the following:

- Monitor and record all AEs.
- Determine seriousness, severity, and relationship to IP and/or study procedure and/or underlying disease.
- Determination of the onset and end date of each event.
- Provide initial report on all SAEs within 24 hours of first knowledge to the Sponsor via email to [redacted] or designee.
- Provide follow-up information on SAEs in a timely and proactive manner.
- Respond to queries regarding AEs and SAEs in a timely manner.
- Ensure source documentation for all AEs are accurate and complete.
- Ensure that the study is conducted as defined in this protocol.

Investigators may also report improvement of pre-existing DMD conditions or unexpected therapeutic responses.

6.3.8. Responsibilities of the Sponsor

The responsibilities of the Sponsor (Sarepta and designees) include, but are not limited to the following:

- Training of Investigator and site staff on AE/SAE definitions, safety assessments, and site obligations related to safety monitoring and reporting of AE/SAEs.
- Accurate and timely reporting of SUSARs to all applicable regulatory bodies, clinical trial Investigators, and other parties as appropriate and required within the regulated timelines.

- Ensuring accurate recording of AEs and SAEs.
- Notification of expedited SUSARs to sites.
- Annual safety reporting to regulatory authorities and IRBs/IECs according to regional requirements.

6.4. Treatment Overdose

For this study, any dose of SRP-9001 above the assigned number of vials for a specific patient will be considered an overdose.

There is no recommended specific treatment for an overdose.

In the event of an overdose, the Investigator should:

1. Contact the CRO's unblinded Medical Director immediately.
2. Closely monitor the participant for any AE/SAE and laboratory abnormalities until resolution.
3. Document the quantity of the excess dose as well as the duration of the overdose on the Overdose Form and send to the Sponsor immediately. The overdose should also be entered in the CRF.
4. Provide appropriate medical care to patient based on clinical judgement.

Decisions regarding dose interruptions or modifications will be made by the Investigator in consultation with the CRO's unblinded Medical Director based on the clinical evaluation of the participant.

6.5. Pharmacokinetics

Pharmacokinetic parameters are not being evaluated in this study.

6.6. Pharmacodynamics

Creatine kinase levels will be measured at time points according to the SoA (Section 1.3). Muscle biopsies will be performed at Screening/Baseline and at Week 12 in Part 1 [redacted].

6.7. [redacted]

[redacted]

6.7.1. [redacted]

[redacted]

[redacted]

[redacted]

6.7.2. [redacted]

[redacted]

[redacted]

[redacted]

6.8. Biomarkers

The collection of muscle biopsy samples and serum blood samples to assess micro-dystrophin and [redacted] will be obtained; however, they are considered efficacy endpoints and described in Section 6.1.

[redacted] [redacted]

[redacted] [redacted]

[redacted]

- [redacted]
- [redacted]
- [redacted]
- [redacted]
- [redacted]

[redacted]

[redacted]

- [redacted]
- [redacted]
- [redacted]
- [redacted]
- [redacted]

[redacted] [redacted]

[redacted]

7. STATISTICAL CONSIDERATIONS

7.1. Statistical Hypotheses

The study has 2 primary endpoints: change in quantity of micro-dystrophin protein expression from Baseline to Week 12 (Part 1) as measured by Western blot and change in NSAA total score from Baseline to Week 48 (Part 1). Each primary endpoint will be tested for statistical significance based on a multiplicity-adjusted testing procedure that will control the overall Type I error rate at a 2-sided level of 0.05. The 2-sided alpha of 0.05 will first be split with 0.01 allocated to the primary endpoint of change in quantity of micro-dystrophin protein expression from Baseline to Week 12 (Part 1) as measured by Western blot, and 0.04 allocated to the primary endpoint of change in NSAA total score from Baseline to Week 48 (Part 1). If a hypothesis is rejected at the allocated alpha, then the allocated alpha can be recycled for testing the other hypothesis. Additional details will be specified in the Statistical Analysis Plan (SAP). The statistical analyses of endpoints in Part 2 will also be described in the SAP.

For the primary endpoint of change in quantity of micro-dystrophin protein expression from Baseline to Week 12 as measured by Western blot, the null hypothesis is that treatment assignment has no effect on the endpoint, and the alternative hypothesis is that treatment assignment affects the endpoint. Even though the alternative hypothesis is 2 -sided, only superiority of SRP-9001 over placebo will be of interest.

The statistical hypotheses for other micro-dystrophin expression endpoints can be stated in a similar manner as those for change in quantity of micro-dystrophin protein expression from Baseline to Week 12 as measured by Western blot.

For the primary efficacy endpoint of change in NSAA total score from Baseline to Week 48 (Part 1), the null hypothesis is that the population means for the 2 treatments are equal and the alternative hypothesis is that the population means for the 2 treatments are not equal. Even though the alternative hypothesis is 2-sided, only superiority of SRP-9001 over placebo will be of interest.

The statistical hypotheses for secondary endpoints on physical function can be stated in a similar manner as those for change in NSAA total score from Baseline to Week 48 (Part 1).

7.2. Sample Size Determination

A dramatic treatment difference is expected for the primary endpoint of change from Baseline to Week 12 in quantity of micro-dystrophin protein expression as measured by Western blot. Therefore, the sample size is based on the power for the primary efficacy endpoint of change in NSAA total score from Baseline to Week 48 (Part 1) with Type I error of 0.05 (2-sided), assuming the alpha allocated for the other primary endpoint will be recycled. Assuming the standard deviation is 5 for this endpoint for the baseline age of 4 to 7, with a Type I error of 0.05 (2-sided), a sample size of 22 patients per treatment group will provide approximately 90% power to detect a mean treatment difference of 5 in change in NSAA total score from Baseline to Week 48. If the sample size is smaller, 17 patients per treatment group will provide approximately 80% power to detect a mean treatment difference of 5 in change in NSAA total score from Baseline to Week 48 with Type I error of 0.05 (2-sided).

7.3. Analysis Populations

For purposes of statistical analyses, the following analysis populations are defined:

Population	Description
Intent-to-Treat (ITT)	All randomized patients who received study treatment during Part 1, with treatment group designated according to randomization. The ITT population will be the main analysis population for endpoints for Part 1.
Per-Protocol	All patients in the ITT population who do not have important protocol deviations that may substantially affect the study results. Eligibility for the Per-Protocol population will be determined prior to breaking the study blind.
Safety	All patients who received study treatment during Part 1 or Part 2, with treatment group designated according to the treatment that they actually received.

7.4. Statistical Analyses

An SAP will be developed and finalized before database lock and unblinding of the study data. It will describe the details of the statistical analyses. This section is a summary of the planned statistical analyses of the primary and secondary endpoints as well as the safety analyses.

7.4.1. Efficacy Analyses

The study has 2 primary endpoints: change in quantity of micro-dystrophin protein expression from Baseline to Week 12 (Part 1) as measured by Western blot and change in NSAA total score from Baseline to Week 48 (Part 1). Each primary endpoint will be tested for statistical significance based on the multiplicity-adjusted testing procedure that will control the overall Type I error at a 2-sided level of 0.05.

Endpoint	Statistical Analysis Methods
Primary	For the primary endpoint of change in quantity of micro-dystrophin protein expression from Baseline to Week 12 (Part 1) as measured by Western blot, summary statistics will be provided by treatment group for Baseline, Week 12, and change from Baseline to Week 12 for quantity of micro-dystrophin protein expression as measured by Western blot.<br>A re-randomization test will be performed to test the null hypothesis that treatment assignment has no effect on change in quantity of micro-dystrophin protein expression from Baseline to Week 12 as measured by Western blot. The superiority of SRP-9001 over placebo will be concluded if the re-randomization test achieves statistical significance based on the multiplicity-adjusted testing procedure that will be specified in the SAP.<br>For the primary efficacy endpoint of change in NSAA total score from Baseline to Week 48 (Part 1), summary statistics will be provided by treatment group for Baseline, each post-Baseline visit, and change from Baseline to each post-Baseline visit.<br>A mixed model with repeat measures or another test deemed appropriate, to be specified in the SAP, will be performed to compare the treatment groups for change

Endpoint	Statistical Analysis Methods
	in NSAA total score from Baseline to Week 48 (Part 1). The superiority of SRP-9001 over placebo will be concluded if the test achieves statistical significance based on the multiplicity-adjusted testing procedure that will be specified in the SAP.
Secondary	The secondary endpoints will be analyzed using similar statistical methods as the primary endpoints, as appropriate, with details specified in the SAP.
[redacted]	[redacted]

7.4.2. Safety Analyses

Four sets of safety analyses will be performed: (1) Part 1, this will provide summaries of safety endpoints in a side-by-side comparison between the two treatment groups that should be similar at Baseline; [redacted] (3) Part 1 [redacted], this will provide summaries of safety endpoints in a side-by-side comparison between the two treatment groups through Part 1 [redacted]; (4) Time since SRP-9001 treatment, this will provide summaries of safety endpoints across all patients who received SRP-9001 in either Part 1 [redacted], starting from the time of SRP-9001 infusion and continue through the end of the study.

Endpoint	Statistical Analysis Methods
Safety: TEAEs, and SAEs	Adverse events will be coded by the Medical Dictionary for Regulatory Activities. TEAEs include all AEs that occurred or increased in severity since the study treatment. A treatment-related TEAE will be defined as a TEAE that the Investigator considered possibly/probably or definitely related to the study treatment.<br>TEAEs will be summarized by treatment group by the number and percentage of patients who reported TEAEs, with grouping by system organ class and preferred term. SAEs will be summarized in the same way. TEAEs will also be summarized by treatment group, with grouping by system organ class, preferred term, and severity. Further, treatment-related TEAEs will be summarized in the same way as TEAEs.
Safety: Clinical laboratory assessments	Clinical laboratory variables will be presented in 3 ways.<br>First, change from Baseline to each scheduled assessment will be summarized descriptively by treatment group. Baseline will be defined as the value of the clinical laboratory variable obtained immediately before dosing of study drug (Day −1 for Part 1 and Day -1 for Part 2). For Part 1, if Day −1 value is unavailable, the value obtained at the Screening Visit will be used.<br>Second, treatment-emergent potentially clinically significant (PCS) laboratory values will be identified. Criteria for PCS laboratory values will be defined in the SAP. Treatment-emergent PCS laboratory values will be defined as post-Baseline PCS laboratory values for patients whose baseline value for the same laboratory variable did not meet the PCS criteria. The number and percentage of patients with a treatment-emergent PCS laboratory value will be summarized by treatment group for each clinical laboratory variable.

Endpoint	Statistical Analysis Methods
	Third, the number and percentage of patients with abnormal, clinically significant laboratory values (per Investigator judgment) will be summarized by treatment group for each clinical laboratory variable.

7.5. Interim Analyses

Up to 4 planned interim analyses may be performed. The SAP will describe the planned interim analyses in greater detail, including the testing procedure to adjust for multiplicity to control the overall Type I error rate at a 2-sided level of 0.05.

7.5.1. Interim Analysis at Week 12 in Part 1

An interim analysis for micro-dystrophin expression will be performed after the blinded assays for micro-dystrophin expression for Baseline and Week 12 (Part 1) have been completed. Unless an unexpected outcome occurs at the interim analysis, there is no plan to change the conduct of the study after the interim analysis. A procedure will be put in place prior to unblinding of any personnel to micro-dystrophin expression data to ensure that the study continues to be conducted as a double blinded study after this interim analysis.

7.5.2. Interim Analysis at the Completion of Part 1

An interim analysis will be performed after all patients have completed Part 1 of the study. This will be the primary analysis of the study. A procedure will be put in place prior to unblinding of a limited number of personnel to ensure that the study continues to be conducted as a double blinded study after this interim analysis.

[redacted]

[redacted]

7.5.4. Interim Analysis Based on Regulatory Interactions

An additional interim analysis may be performed based on regulatory interactions. The decision to perform this interim analysis may take into account information external to this study and/or blinded study data review. If this interim analysis is to be performed during Part 1 or Part 2, a procedure will be put in place prior to this interim analysis to ensure that the study continues to be conducted as a double blinded study after this interim analysis.

7.6. Data Monitoring Committee

An independent DMC will be established to periodically review the safety data of this study and provide recommendations to the Sponsor. The DMC will operate under a DMC charter to be approved by the DMC members and the Sponsor.

8. DISCONTINUATION OF STUDY INTERVENTION AND PARTICIPANT DISCONTINUATION/WITHDRAWAL

8.1. Discontinuation of Study Intervention

Participants will receive a single-dose of study treatment in Part 1 and Part 2 of the study; therefore, discontinuations from the study intervention are not anticipated.

8.2. Participant Discontinuation/Withdrawal from the Study

- A participant may withdraw from the study at any time at his own or legal guardian's request or may be withdrawn at any time at the discretion of the Investigator for safety, behavioral, compliance, or administrative reasons. This is expected to be uncommon.
- At the time of discontinuing from the study, if possible, an early termination visit should be conducted, as shown in the SoA (Section 1.3). See the SoA (Section 1.3) for data to be collected at the time of study discontinuation and follow-up and for any further evaluations that need to be completed.
- If the participant (or legal guardian) withdraws consent for disclosure of future information, the Sponsor may retain and continue to use any data collected before such a withdrawal of consent.
- If a participant withdraws from the study, he (or legal guardian) may request destruction of any samples taken and not tested, and the Investigator must document this in the site study records.

8.3. Lost to Follow-up

A participant will be considered lost to follow-up if he repeatedly fails to return for scheduled visits and is unable to be contacted by the study site.

The following actions must be taken if a participant fails to return to the clinic for a required study visit:

- The site must attempt to contact the participant (or if applicable, the legal guardian who signed the informed consent on behalf of the participant) and reschedule the missed visit as soon as possible and counsel the participant (or if applicable, the legal guardian who signed the informed consent on behalf of the participant) on the importance of maintaining the assigned visit schedule and ascertain whether or not the participant wishes to and/or should continue in the study.
- Before a participant is deemed lost to follow up, the Investigator or designee must make every effort to regain contact with the participant (or if applicable, the legal guardian who signed the informed consent on behalf of the participant) (where possible, 3 telephone calls and, if necessary, a certified letter to the participant's last known mailing address or local equivalent methods). These contact attempts should be documented in the participant's medical record.

- Should the participant continue to be unreachable, he/she will be considered to have withdrawn from the study.

9. SUPPORTING DOCUMENTATION AND OPERATIONAL CONSIDERATIONS

9.1. Appendix 1: Regulatory, Ethical, and Study Oversight Considerations

9.1.1. Clinical Monitoring of the Study

The study will be monitored in compliance with the relevant parts of 21 Code of Federal Regulations (CFR) and according to the International Council on Harmonisation (ICH) Good Clinical Practices (GCP) Guidelines. The procedures outlined in the protocol and case report forms will be carefully reviewed by the PI and staff prior to study initiation to ensure appropriate interpretation and implementation. No deviations from the protocol shall be made except in emergency situations where alternative treatment is necessary for the protection, proper care and wellbeing of patients.

9.1.2. Regulatory and Ethical Considerations

- This study will be conducted in accordance with the protocol and with the following:
  - Consensus ethical principles derived from international guidelines including the Declaration of Helsinki and Council for International Organizations of Medical Sciences International Ethical Guidelines
  - Applicable ICH GCP Guidelines
  - Applicable laws and regulations
- The protocol, protocol amendments, ICF, Investigator Brochure, and other relevant documents (eg, advertisements) must be submitted to an IRB/IEC by the Investigator and reviewed and approved by the IRB/IEC before the study is initiated.
- Any amendments to the protocol will require IRB/IEC approval before implementation of changes made to the study design, except for changes necessary to eliminate an immediate hazard to study participants.
- The Investigator will be responsible for the following:
  - Providing written summaries of the status of the study to the IRB/IEC annually or more frequently in accordance with the requirements, policies, and procedures established by the IRB/IEC
  - Notifying the IRB/IEC of SAEs or other significant safety findings as required by IRB/IEC procedures
  - Providing oversight of the conduct of the study at the site and adherence to requirements of 21 CFR, ICH guidelines, the IRB/IEC, European regulation 536/2014 for clinical studies (if applicable), and all other applicable local regulations

9.1.3. Financial Disclosure

Investigators and sub-Investigators will provide the Sponsor with sufficient, accurate financial information as requested to allow the Sponsor to submit complete and accurate financial

certification or disclosure statements to the appropriate regulatory authorities. Investigators and sub-Investigators are responsible for providing information on financial interests during the course of the study and for 1 year after completion of the study.

9.1.4. Informed Consent Process

Legally effective and properly executed written informed consent, in compliance with 21 CFR 50 and the ICH guidelines, will be obtained from each patient, or legal guardian where applicable, before the patient is entered into the trial or before any unusual or non-routine procedure is performed that involves risk to the patient. The informed consent will be signed prior to study procedures and will require IRB approval. Attention will be directed to the basic elements that are required for incorporation into the informed consent under United States Federal Regulations for Protection of Human Subjects [21CFR 50.25(a)]. The final IRB-approved document as well as any subsequent approved modified consent document(s) must be provided to correspondent agencies for regulatory purposes. If new information related to the study arises, patients, or legal guardians where applicable, will be asked to sign a revised document. Signed consent forms will remain in each patient's research chart and will be available for verification by study monitors at any time. Patients, or legal guardians where applicable, will be given a signed, dated copy of their consent form.

- The Investigator or his/her representative will explain the nature of the study to the participant or his legally authorized representative and answer all questions regarding the study.
- Participants, and legal guardians where applicable, must be informed that their participation is voluntary. Participants or their legally authorized representative will be required to sign a statement of informed consent that meets the requirements of 21 CFR 50, local regulations, ICH guidelines, Health Insurance Portability and Accountability Act requirements, where applicable, and the IRB/IEC or study center.
- The medical record must include a statement that written informed consent was obtained before the participant was enrolled in the study and the date the written consent was obtained. The authorized person obtaining the informed consent must also sign the ICF.
- Participants must be re-consented to the most current version of the ICF(s) during their participation in the study.
- A copy of the ICF(s) must be provided to the participant or the participant’s legally authorized representative.

The ICF will contain a separate section that addresses the use of remaining mandatory samples for optional exploratory research. The Investigator or authorized designee will explain to each participant the objectives of the exploratory research. Participants, and their legal guardians where applicable, will be told that they are free to refuse to participate and may withdraw their consent at any time and for any reason during the storage period.

9.1.5. Data Protection

9.1.5.1. Data Safety Monitoring Plan

All data and observations will be documented on CRFs by source documentation. A Study Monitor will have access to the data to monitor adherence to the protocol and to applicable regulations, and the maintenance of adequate and accurate clinical records. A CRF will be completed for every patient that was randomized for participation in the study. The CRFs will be completed as data is generated. Additional information can be found in the CRF Completion Guidelines.

9.2. Committees Structure

9.2.1. Data Monitoring Committee

The independent DMC will act in an advisory capacity to review participant safety and study progress for the clinical trial. For additional information, refer to the Study Operations Manual and DMC Charter.

9.2.2. Dissemination of Clinical Study Data

9.2.2.1. Final Study Report

The final study report will include data through the final study visit.

9.2.3. Source Documents

- Source documents provide evidence for the existence of the participant and substantiate the integrity of the data collected. Source documents are filed at the Investigator's site.
- Data reported on the CRF or entered in the electronic CRF that are transcribed from source documents must be consistent with the source documents or the discrepancies must be explained. The Investigator may need to request previous medical records or transfer records, depending on the study. Also, current medical records must be available.

9.2.4. Publication Policy

The information that is developed during the conduct of this clinical study is considered to be strictly confidential. This information may be disclosed only as deemed necessary by Sarepta Therapeutics, Inc. At the conclusion of this clinical study, a clinical study report will be prepared. In addition, a manuscript may be prepared for publication in a reputable scientific journal under the direction of Sarepta. Sarepta Therapeutics, Inc. will publish and communicate the clinical study results, irrespective of positive or negative findings. Data generated for this study will be exclusively owned by Sarepta Therapeutics, Inc., as detailed in the Clinical Trial Agreement. The study will be registered on ClinicalTrials.gov. After completion of the study, results will be disseminated through ClinicalTrials.gov.

9.3. Appendix 2: [redacted]

[redacted]

10. REFERENCES

[redacted]

Brooke MH, Fenichel GM, Griggs RC, et al. Clinical investigation in Duchenne dystrophy: 2. Determination of the "power" of therapeutic trials based on the natural history. Muscle Nerve. 1983;6(2):91-103

D'Amario D, Amodeo A, Adorisio R, et al. A current approach to heart failure in Duchenne muscular dystrophy. Heart. 2017;103(22):1770-1779.

Eagle M, Baudouin SV, Chandler C, et al. Survival in Duchenne muscular dystrophy: improvements in life expectancy since 1967 and the impact of home nocturnal ventilation. Neuromuscul Disord. 2002;12(10):926-929.

[redacted]

Mazzone E, Martinelli D, Berardinelli A, et al. North Star Ambulatory Assessment, 6-minute walk test and timed items in ambulant boys with Duchenne muscular dystrophy. Neuromuscul Disord. 2010;20(11):712-716.

Mendell JR, Campbell K, Rodino-Klapac L, et al. Dystrophin immunity in Duchenne's muscular dystrophy. N Engl J Med. 2010;363(15):1429-1437.

[redacted]

Signature: [redacted]

Electronically signed by: [redacted]
Reason: I approve this document.
Date: Aug 28, 2020 11:41 EDT

Email: [redacted]

Title:

Company: Sarepta Therapeutics Inc
