# Supplementary material for: Validity of remote live stream video evaluation of the North Star Ambulatory Assessment in patients with Duchenne muscular dystrophy
Source: PLoS One. 2024 May 16;19(5):e0300700. doi: 10.1371/journal.pone.0300700 (PMC11098514; doi:10.1371/journal.pone.0300700)
Supplement: S1 File — (DOCX) [file pone.0300700.s007.docx]

**CONSENT TO PARTICIPATE IN A CLINICAL RESEARCH STUDY**

**STUDY TITLE:** Systemic gene delivery Phase I/IIa clinical trial for Duchenne muscular dystrophy using rAAVrh74.MHCK7.micro-dystrophin (microDys-IV-001)

**PROTOCOL NUMBER AND VERSION:** SRP-9001-101, Version 9.0 (Amendment 8) 25 Aug 2020

**PRINCIPAL INVESTIGATOR:** Jerry Mendell, M.D.

**CONTACT TELEPHONE NUMBER:** 614-843-4496 (24 hours a day, 7 days a week)

**STUDY SPONSOR:** Sarepta Therapeutics

**SUBJECT’S NAME:**

**DATE OF BIRTH:**

**NOTE: The words “you” and “your” are used in this consent form. These words refer to the**

**study volunteer whether a child or an adult**

**Key Information About This Study**

The following is a short summary of this study to help you decide whether or not to participate. More detailed information follows later in this form.

The purpose of this study is to try to find out if it is safe to deliver your missing dystrophin gene through the blood stream with a new one. This can be done by a method called “gene transfer.” Doctors will transfer a smaller version of the dystrophin gene (called micro-dystrophin) made in the laboratory and put it into your body.

Study participation:

Subjects will have the following over the duration of the study: physical exams, vitals, EKGs, ECHOs, MRIs, a chest X-ray, blood collection, urinalyses, physical therapy assessments, and muscle

biopsies. The gene transfer will occur through an IV over 1-2 hours.

Study visits:

This study will require up to 19 visits to Nationwide Children’s Hospital. These visits will be spread

over five years. During this time, you will first have an outpatient screening visit to make sure that it is safe to undergo gene transfer. Next, you will be admitted to the hospital for the gene transfer

procedure. You will stay approximately 48 hours. Finally, there will be outpatient follow-up visits to

make sure that there are no side effects. During one of these follow-up visits you will have a muscle biopsy. The study will last for five years. Unscheduled visits or labs may be added if the PI feels that they are needed for safety.

See a more detailed discussion later in this form.

The main risks of the study are reaction to the gene transfer, side effects associated with steroid

treatment and anesthesia, and complications from the muscle biopsy procedure. See the complete list of risks described later in this form. Other risks are listed later in this form.

There may be no direct benefit to you from being in this study, but the Study Doctors might learn something that could help others.

If you are interested in learning more about this study, please continue reading below.

**1) INTRODUCTION**

We invite you to be in this research study because he has a muscle disorder called Duchenne muscular dystrophy (DMD). The Study Doctor is investigating the safety of a drug thought to help make a protein that you, and other patients who have DMD, do not make. This missing protein is called dystrophin. Microdystrophin is a smaller form of the dystrophin protein that your muscles are missing. You will need to learn enough about this research study and its risks and benefits to decide whether you should agree to participate. This process is called “informed consent”.

Participation is voluntary. The Study Doctor must explain the study to you and give you a chance to ask questions about anything you do not understand. Using this form as a guide, we will explain the study to you. If you have any questions about the study, please ask. By signing this form, you agree to be in this study. If you do not want to be in this study, all regular and standard medical care will still be available to you here at Nationwide Children’s Hospital. Participation is voluntary. You can leave this study at any time.

You will be given a signed and dated copy of the consent form.

Sarepta Therapeutics, Inc., is the sponsor of this research study. Sarepta will pay the study center,

Nationwide Children’s Hospital, to cover the costs of conducting this research study.

**2) WHERE WILL THE STUDY BE DONE AND HOW MANY SUBJECTS WILL TAKE PART?**

This study will be done at one site, Nationwide Children’s Hospital. Overall, twelve (12) participants were planned to take part in this study; however, the study will only enroll 4 participants. Cohort A was planned to include six subjects ages 3 months to 3 years. Cohort B will include subjects ages 4 to 7 years. All subjects will receive the study drug. Cohort B will be enrolled before Cohort A. Eligibility will be based on the following criteria:

Subjects must meet the following criteria:

• Age of enrollment: Cohort A (n=6) is between 3 months to 3 years of age, inclusive; Cohort B (n=6) is between 4 to7 years of age, inclusive.

• Molecular characterization of the DMD gene with frameshift (deletion or duplication), or premature stop codon mutation between exons 18 to 58.

• Indication of symptomatic muscular dystrophy:

o CK elevation >1000 U/L **and**

o Cohort A: below average on the Bayley-III motor assessment for gross motor defined

as a scaled score of ≤9. Any subject that is 43-47 months of age, inclusive, at time of

screening will have the scaled score calculated compared to normative data for 42

month old children. The Bayley-III provides normative data for children 1-42 months of age.

o Cohort B: below average on the 100 Meter Timed Test defined as ≤ 80% predicted.

• Males of any ethnic group will be eligible.

• Ability to cooperate with motor assessment testing.

• For Cohort A subjects: No previous treatment with corticosteroids. For Cohort B subjects: Stable dose equivalent of oral corticosteroids for at least 12 weeks prior to screening and the dose is

expected to remain constant (except for modifications to accommodate changes in weight)

throughout the first year of the study.

Subjects meeting the following criteria will be excluded from the study:

• Active viral infection based on clinical observations.

• Signs of cardiomyopathy, including echocardiogram with ejection fraction below 40%.

• Serological evidence of HIV infection, or Hepatitis B or C infection.

• Diagnosis of (or ongoing treatment for) an autoimmune disease.

• Abnormal laboratory values considered clinically significant (GGT > 3XULN, bilirubin ≥ 3.0 mg/dL, creatinine ≥ 1.8 mg/dL, Hgb < 8 or > 18 g/Dl; WBC > 18,500 per cmm).

• Concomitant illness or requirement for chronic drug treatment that in the opinion of the PI

creates unnecessary risks for gene transfer.

• Subjects with AAVrh74 or AAV8 antibody titers > 1:400 as determined by ELISA

immunoassay.

o If endpoint titer is positive testing may be repeated prior to exclusion.

o If present in infant and mother is positive for same antibody titers, mother will be asked

not to breast feed and infant can be enrolled if antibodies drop ≤1:400 within 12 weeks.

• Has a medical condition or extenuating circumstance that, in the opinion of the investigator, might compromise the subject’s ability to comply with the protocol required testing or procedures or compromise the subject’s wellbeing, safety, or clinical interpretability.

• Severe infection (e.g., pneumonia, pyelonephritis, or meningitis) within 4 weeks before gene transfer visit (enrollment may be postponed).

• Has received any investigational medication (other than corticosteroids) or exon skipping medications, experimental or otherwise, in the last 6 months prior to screening for this study.

• Has had any type of gene therapy, cell based therapy (e.g. stem cell transplantation), or

CRISPR/Cas9.

• Family does not want to disclose patient’s study participation with primary care physician and other medical providers.

**3) WHAT WILL HAPPEN DURING THE STUDY AND HOW LONG WILL IT LAST?**

This study will require up to 18 visits to Nationwide Children’s Hospital. These visits will be spread over five years. During this time, you will first have an outpatient screening visit to make sure that it is safe to undergo gene transfer. Next, you will be admitted to the hospital for the gene transfer procedure. You will stay approximately 48 hours. Finally, there will be outpatient follow-up visits to see if there are any side effects. During one of these follow-up visits you will have a muscle biopsy. The study will last for five years. Unscheduled visits or labs may be added if the Study Doctor feels that they are needed for safety. See Appendix 1 (last page of this document) for a study timeline.

Visit 1 - Screening Visit:

To begin the study, you will come for a screening visit to be sure you understand what is involved. The study team will also decide if it is safe for you to be in this study. You will meet the Study Doctor and the study staff. Before this visit, you will receive a “voided copy” of the consent. This voided form

is just a copy of the actual consent form that you will sign when you come to the screening visit. We encourage you to read it carefully and discuss it with your family and your doctor. Reading this form in advance will give you the chance to get familiar with the study and prepare any questions you may want to ask the Study Doctor. During the screening visit, the study staff will explain exactly what it means to be a part of this study and answer all of your questions. If you would like more time to consider the study before making your decision, you can go home and come back on another day. You will be given as much time as you would like to discuss this study with your family or primary care doctor. You are encouraged to do this.

Once you are satisfied with all of the answers to your questions, and you have decided to have you be part of this study, you will have a full exam that will take about half of a day. The exam will include questions about your medical history, past and present illnesses, allergies and medications. It is very important that you tell the Study Doctor your complete medical history and any medications you are taking. You will be encouraged to maintain their medications, vitamins and supplements they are on at enrollment through the course of the study, but you need to keep the research team informed of any changes on the list of medications in every follow up visit.

If you have an inflammatory muscle or bowel disease, you may not be allowed to be in the study since these illnesses and their treatment can weaken the immune system and make you more likely to have reactions against the gene transfer.

Study assessments:

• You will be asked to give a urine sample in a cup.

• You will also have blood drawn from your arm (about 10 teaspoons) that will undergo standard tests in the hospital lab and some experimental tests in our research laboratory.

• You will also have an electrocardiogram (ECG) and an echocardiogram (ECHO). For the ECG, you will have leads put on your chest, arms, and legs. This will make a graph of your heartbeat.

An ECHO sends sound waves to your heart and makes an image.

• You will also have an x-ray of their chest and an MRI of your heart. An MRI (Magnetic

Resonance Imaging) uses magnets to take a picture of something inside your body.

• In addition to these tests, a physical therapist will test your muscle function by performing various assessments.

• Finally, you will have a pre-treatment muscle biopsy. The biopsy will be done in a leg muscle.

This will be compared to a biopsy that is performed later in the study.

Your functional assessments will be videotaped for quality control so that researchers can be sure that the study assessments are being performed properly. These videos will be reviewed for research purposes by study staff at your site, the Sponsor or evaluators contracted by the Sponsor, and may be viewed by government agencies if the study goes through a quality review. These videos may also be used to demonstrate any functional outcomes related to the study drug and shared with regulatory authorities. Your name or other identifying personal information will not be included with these videos. However, it is possible that someone may recognize you from the images. You will not receive, and are giving up any claim to receive, any payment or royalties in connection with the use of the recorded videos.

Gene Transfer: Day -1 to Day 0:

• You will be admitted to the hospital the day before the gene transfer. One person can stay with you in the room before the procedure.

• A physical exam will be done by one of the study doctors.

• You may receive a medicine to help them calm down before the procedure if you feel nervous.

The Study Doctor will decide if it is necessary to give this medicine either by mouth or through an Intravenous Line (IV).

• You will get an IV placed. This is done by placing a needing through the skin into a vein in the hand or arm. The needle is then taken out leaving a small soft tube known as a catheter. The

catheter will allow the doctors and staff to give you the study drug by gene transfer.

• You will have blood drawn on Day -1. A urine sample will also be collected.

• The gene transfer will only occur on this day and will not be given a second time during the study.

• Pictures of the injection site will be taken before and after the procedure.

• After the procedure, the nurses will make sure you feel okay. For the remainder of the day and night the nurses and study team will make sure that you have no bad reactions (such as fever or vomiting).

• If you feel well the next morning, then you can be discharged from the hospital after the Study

Doctor examines you a final time.

Follow-up Visits: Day 7 through 5 years:

You will be asked to return to Nationwide Children’s Hospital to be seen on Days 7, 14 , 30, and 60 and months 3, 6, 9, 12, 18, 24, 30, 36, 42, 48, 54, and 60. You may also be asked to return for a short visit

1-2 weeks after the biopsy at Day 90.

The following assessments will be completed at every follow-up visit:

• Vitals

• Physical Exam

• Pictures of the Infusion Site

• Safety blood and urine tests (approximately 17 mL/3.5 tsp. of blood will be collected during visits)

• Research immunology tests from blood

The following assessments will only be completed at some of the follow-up visits (refer to the study timeline):

• ECHO/EKG

• MRI

• PT Assessments

• Muscle Biopsy

• Pictures of the Injection Site

The muscle biopsy will be performed on Day 90. You will not have to stay in the hospital overnight. A

biopsy is a minor operation where a small amount of muscle (about the size of two tic-tacs) is taken. The leg muscle will be biopsied. Over this area, the skin will be cleaned with a solution to prevent

infection (something like iodine unless you are allergic). You will receive an injection in the skin to numb

the area and a very small incision will be made less than 1 centimeter long. The Study Doctor or another doctor skilled in muscle biopsies will perform the biopsy. Your ability to move or function will not be affected by the removal of this muscle. Once the muscle tissue is removed it will be compared to the biopsy taken before the gene transfer. It will be tested to check the size of the muscle cells, check the level of dystrophin found in the muscle, and to see if your body reacted to the study drug. The muscle tissue taken from the screening biopsy will be used as a comparison.

After the biopsy, you can either return to our center or have the stitches removed at a local clinic. If you return to our clinic, then pictures will be taken as well as blood and urine tests.

Some tests may provide information that we were not specifically looking for in this study. This information is called “incidental findings”. We will discuss these results with you if we believe that they may have a significant impact on your health or family’s health. If you ask us to do so, we can also help you set up follow-up meetings with your regular doctor or other medical professionals not involved in this study who can discuss this information with you. These follow-up visits will not be part of this study. Therefore, you and your insurance company would be responsible for any fees and costs related to them.

Your samples and information will be used only for research and will not be sold. You will not receive any money or other compensation for any new products that might be developed or sold from this research.

**4) WHAT ARE THE RISKS OF BEING IN THIS STUDY?**

It is important that you give the study staff a COMPLETE medical history. Not giving this information or not completely following the directions of the study team could harm you.

All study medications may cause some side effects or other reactions. The side effects and discomforts most commonly associated with gene transfer and procedures used in this study are listed below. There is no way to predict if you will experience any of these side effects.

**Risks of SRP-9001**

The study drug is still being studied so the Sponsor does not know all the risks your child may experience. Studies on animals that received larger IV doses showed no side effects that were directly related to the study drug. There is a small chance that the micro-dystrophin gene could damage normal genes in your child’s muscle. There is a potential complication of increased cancer risk with gene transfer. However, this complication is unlikely as complications from spread of virus or micro-dystrophin gene to other body parts in animal studies have not shown the development of cancer in treated animals.

Your child may have an immune reaction to the gene therapy. This is called an immune response which is like an allergic reaction. In the unlikely scenario that your child has elevated rAAVrh74 antibodies at the time of screening which were not detected (“false negative”), the risk of these reactions may be increased.

Some immune reactions can occur during or shortly after infusion. These could cause itching, swelling, or redness most likely at the site of injection but could also cause generalized swelling and shortness of breath or even death. So far, there have been no serious or severe immune reactions during or shortly after infusion with SRP-9001, or deaths at any time.

Some other immune reactions are delayed (they appear after a few days to weeks) and can include muscular weakness, which may lead to difficulty walking, swallowing, and breathing; and/or rashes, decrease in blood cell count, joint pain, or damage to the kidney and blood vessels, or damage to the liver. So far, SRP-9001 has caused liver damage in some patients (described below), and muscular weakness in one patient, and these may be immune reactions. The other types of immune reactions (rashes, decrease in blood cell count, joint pain, damage to the kidney and blood vessels) have been observed as reactions to other therapies, but have not yet been seen in reaction to SRP-9001.

In the unlikely scenario that your child has elevated rAAVrh74 antibodies at the time of screening which were not detected (“false negative”), the risk of these reactions may be increased. The only identified reactions to the study drug so far which may be an immune response are liver damage described below, and one instance of muscular weakness. An immune response which leads to problems like those above may need to be treated with medications that dampen the immune response, or with plasmapheresis. Plasmapheresis is a procedure that removes blood plasma from a person's body, treats it to remove any present antibodies, and then returns the treated plasma to the person's body.

One of the blood tests your child will have will test for the body’s reaction to the vector. With or without a reaction to the study drug used in this trial, participation in this study may prevent your child from participating in a future gene transfer trial using this vector to deliver the gene. This is because, once exposed, the body will likely develop antibodies to the vector, which may prevent this vector from being as effective and/or safe a second time. However, treatment might be possible with a different vector or by using a different way to deliver the gene.

There is a possible risk of liver damage. Viral vector gene transfer is likely to cause elevations in certain liver enzymes, resulting in abnormal values in blood tests. These may be temporary, and have not been associated with significant liver injury. However, recent research studies testing somewhat higher doses of adeno-associated virus (AAV) and a different gene than the study drug in experimental animals have raised further concerns about the potential for liver damage, as well as problems with balance and coordination, and even death. Reversible elevations in liver enzymes including severe liver enzyme elevations have occurred in people receiving the study drug. In this study your child will be monitored for side effects.

Some mild or moderate temporary decreases in the number of platelets, which are a kind of blood cell, have been observed in people receiving the study drug in the first week, and these have all recovered on their own. Low platelets may result in easy bruising or bleeding. In this study your child’s platelets will be monitored.

With other gene therapies, a risk of a serious condition called hemolytic uremic syndrome, referred to as “HUS” or “aHUS,” has been observed. This has not yet been seen with SRP-9001. This condition includes a drop in blood counts and kidney injury, and when it has occurred with other gene therapies, it has required emergent hospitalization and treatment. Your child will be monitored for drops in blood counts and for kidney injury, and if these arise, they can be treated.

Some people who have received the study drug have experienced transient intermittent nausea and vomiting in the few weeks after gene transfer. Sometimes these symptoms were treated with anti-nausea medication, and all resolved without any long-term problems.

It is possible that the study drug vector containing the micro-dystrophin gene could interact with other viruses with which your child could come into contact, like cold viruses. If this happens, the study drug vector might form a virus that makes your child sick.

The process to make gene vectors requires the use of biological materials, including cells not from your child and DNA not from your child. Due to the process of making gene vectors, it is possible that pieces of DNA that are not planned to be in the vector may be there after manufacturing.

**For a period of 4 weeks after study drug infusion, people who may come contact with your child’s bodily fluids and waste must regularly wash their hands with soap and people who will have direct contact with your child’s bodily fluids and waste must wear protective gloves. Your child may not donate blood for two years following the study drug infusion. Please refer to the Participant Study Guide for additional information.**

**Risks from Muscle Biopsy**

Potential complications from the muscle biopsy procedure include:

a) Infection at the biopsy site, which may require early stitch removal or possible antibiotic treatment.

b) Bleeding at the site of the biopsy that could require additional stitches.

c) Pain at the site of the biopsy. Acetaminophen (also known as Tylenol®) will be offered every

4 hours as needed to control pain. Please contact the Study Doctor at the number listed on the first page if acetaminophen does not take care of the pain.

d) There will be a scar at the site of the incision; usually this is small but occasionally some

individuals have a tendency to develop bigger scars.

**Risks Associated with Procedures**

You may have pain at the time of injection that could last for a few days. There may be bruising around the injections. You may feel faint at the time of injection. It is unlikely that the injection will cause an infection.

**Risks related to Prednisone/prednisolone (steroids) treatment**

The possible side effects of steroids include:

• acne

• increased hair growth

• thinning of the skin

• glaucoma

• roundness of the face

• changes in behavior

• disturbance of sleep

• weight gain

**Electrocardiogram**

During an ECG, sticky patches are placed on the chest. There may be discomfort or a temporary rash when the patches are removed from the skin like taking off Band-Aid.

**Magnetic Resonance Imaging (MRI)**

There are no known risks to the types of magnetic fields and radio waves used in these studies, but an unknown risk is always possible. Rarely (one in thousands of exams) sunburn-like skin may

appear over a small area of the body but special precautions are taken to prevent this. You must lie very still in a small space during this procedure

**Sedation**

Calming medicine (sedative) may cause temporary side effects such as:

• drowsiness

• shaking

• chills

• dizziness

Rarely this type of medicine may cause:

• unsteadiness

• breathing difficulty

• low blood pressure

• heart rate changes

• allergic reactions

• very rarely, death.

Emergency equipment will be available whenever sedation is required.

The number of times you will be exposed to sedation and anesthesia will depend on their condition and how well they tolerate procedures. Depending on your circumstances, additional monitoring may be required after anesthesia which would require you to stay in the hospital longer than normal or even overnight. The study team will discuss this with you.

The risk of receiving multiple doses of anesthesia in a short period of time is unknown. There is some data regarding children less than 3 years of age that shows multiple doses of anesthesia can have a negative impact on your ability to learn.

**Other Risks**

If you are worried about anything while in this study, please call the study team at the telephone number on page 1 of this form.

Drawing blood and starting IVs by placing a needle in a vein may cause pain, lightheadedness, fainting, bleeding, bruising, clotting, nerve damage or swelling at the puncture site. Infection is a rare possibility. If needed, numbing cream may be used on the skin to decrease the discomfort. Skin irritation or an allergic reaction is possible from the numbing cream.

Your child may experience dark urine caused by muscle breakdown as a result of their disease or possibly due to its treatment with drugs including the study drug.. The darkened urine could indicate a potential of kidney harm and require hospital admission for treatment. Please contact the study doctor should you have any questions, concerns or if your child develops dark urine (described as tea-colored, cola-colored or slightly brown).

Elevation of troponin (a protein normally found in the muscle of the heart) has been described in muscular dystrophy disease and increased troponin levels have been reported in patients with DMD like your child, sometimes accompanied by chest pain. Abnormalities in cardiac function, including elevated troponin, have been observed with the use of other AAV drugs. This has not yet been seen with SRP-9001. In this study your child’s troponin levels will be monitored.

Your child will be closely monitored and treated for any complications during and after the procedure.

The study doctor and team will check closely for side effects with periodic safety tests (for example, blood tests) and physical exams.

Although we will take every precaution, there is a small chance of loss of confidentiality of your child’s study information.

There may be other risks of being in this research study that are not known at this time.

**5) SPECIAL INFORMATION ABOUT SEXUAL INTERCOURSE AND PREGNANCY:**

Pregnancy should be avoided, and an effective method of birth control must be practiced during the whole study. The best way to avoid pregnancy is abstinence (not having sexual intercourse). Talk to the study team about medically approved forms of birth control such as:

• Birth control pills

• Intra Uterine Device (IUD)

• Hormone implants

• Contraceptive Injection

• Barrier Method (diaphragm with spermicidal gel or condoms with contraceptive foam) If at any time, there is a suspicion of pregnancy, you must call the study team right away.

**6) ARE THERE BENEFITS TO TAKING PART IN THIS STUDY?**

.Although there may be no benefit to you from being in this study, we hope to learn something that could help others.

**7) WHAT OTHER TREATMENTS OR OPTIONS ARE THERE?**

Your participation is this study is voluntary. It is not necessary to participate in this study in order for you to get care for your condition.

There is no cure for DMD. One FDA approved treatment is available (called Exondys 51), only for DMD patients with a mutation amenable to the skipping of exon 51. Supportive care and the use of steroids are currently available as well.

While you are enrolled in this trial, we request that you do not take other drugs for treatment of DMD disease other than your corticosteroid drug. This is so we can evaluate whether the gene therapy we are treating you with is safe and working. If you choose to take an exon skipping drug during the course of the trial, you must tell the study doctor as soon as you begin the drug. If you choose to take an exon skipping drug during this trial, all regular and standard medical care will still be available to you here at Nationwide Children’s Hospital. You will be asked to continue your safety visits for the remainder of the trial but your data will no longer be included in the trial outcomes.

**8) WHAT ARE THE COSTS AND REIMBURSEMENTS?**

You will not be paid for being in this study. The cost of all inpatient and outpatient visits, evaluations, procedures, and tests related to the study will be covered by the Sponsor.

**9) WHAT HAPPENS IF BEING IN THIS STUDY CAUSES INJURIES?**

If you are hurt by the Study Doctor or the procedures that are part of this study, you should seek medical treatment for the injuries and tell the Study Doctor as soon as possible at the number on page 1 of this form. If it is an emergency, call 911 or go to the nearest emergency department.

If your injury or illness is the result from Duchenne muscular dystrophy or from standard clinical care, this medical care will be billed to your health insurance company or whoever usually pays for your health care at the usual charges for treatment.

If you are hurt by the study drug or properly performed study procedures and you have followed the directions of the study team, the Sponsor will pay for the medical expenses necessary to treat the injury. Costs of injuries arising from your underlying condition will not be paid for by the Sponsor. The Sponsor will also not pay for things like lost income that are a result of the injury or illness.

In the event the Sponsor provides any reimbursement for medical treatment, the Sponsor must comply with federal reporting requirements relating to the reimbursement of such costs, including providing to Medicare your Health Insurance Claim Number or, if none is available, your social security number. By signing this consent, you agree that if asked by the study team, you will provide such information to the Principal Investigator for disclosure to the Sponsor.

If the care is provided at Nationwide Children's Hospital, we make no commitment to pay for the medical care provided to you. No funds have been set aside to compensate you in the event of an injury. If no one else pays for your care, you may have to pay for the cost of this care. This does not mean that you give up any of your legal rights to seek compensation for your injuries.

**REQUEST FOR AUTOPSY**

Per the National Institutes of Health’s Guidelines for Research Involving Recombinant or Synthetic

Nucleic Acid Molecules (effective March 2013), we will request an autopsy be performed if a study participant dies, no matter what the cause. This is requested to obtain vital information about the safety and efficacy of gene transfer. We ask you advise the members of your family of this request and of its scientific and medical importance. You have the option to decline the autopsy.

A description of this clinical trial will be available on [http://www.ClinicalTrials.gov,](http://www.clinicaltrials.gov/) as required by U.S. Law. This Website will not include information that can identify you. At most, the Website will include a summary of the results. You can search this Web site at any time.

Details of the research study or your progress shall not be discussed with anyone outside of your immediate family, including other study participants or their families that you may be in contact with. You should not discuss your involvement in this study with others, especially on social media sites such as Facebook and Twitter. Doing so could affect the results of the research study. This is prohibited, because at this time we have no definite study outcome and may give others an erroneous impression.

Because this study involves gene transfer, you are expected to:

• participate in long-term follow-up that extends beyond this study;

• provide a back-up contact person(s) for investigator questions; and

• keep a current address and telephone number with the study team.

Nationwide Children’s Hospital is a teaching hospital and we are committed to doing research. Doing research will enable us to learn and provide the best care for our patients and families. You may be asked to participate in other research studies in the future. You have the right to decide to participate or decline to participate in any future studies. We will not share your contact information with researchers outside Nationwide Children’s Hospital.

**10) WHAT WILL HAPPEN IF NEW INFORMATION IS FOUND OUT ABOUT THE DRUG OR TREATMENT?**

If new information is found out during this study that might change your mind about participating or might affect your health, you will be informed of significant findings.

**11) WHAT HAPPENS IF I DO NOT FINISH THIS STUDY?**

It is your choice to be in this study. You may decide to stop being in this study at any time. If you decide to stop being in this study, call the study team at the number on page 1 of this form to see if there are any medical issues about stopping. If you stop being in the study, there will be no penalty or loss of benefits to which you are otherwise entitled.

**12) OTHER IMPORTANT INFORMATION**

It is important that you tell your other doctors about all medicines that you are taking including the medicine being tested in this research study.

Being in more than one research study at the same time may cause injury. Tell us if you are in any other research studies.

While you are participating in this study, you may not be able to get access to your medical records related to this study because it could interfere with the results of the study. As soon as the study is finished, you will have access to these medical records.

A description of this clinical trial will be available on [http://www.ClinicalTrials.gov,](http://www.clinicaltrials.gov/) as required by U.S. Law. This Website will not include information that can identify you. At most, the Website will include a summary of the results. You can search this Web site at any time.

Because this study involves gene transfer, you are expected to:

• participate in long-term follow-up that extends beyond this study;

• provide a back-up contact person(s) for investigator questions; and

• keep a current address and telephone number with the study team.

Nationwide Children’s Hospital is a teaching hospital and we are committed to doing research. Doing research will enable us to learn and provide the best care for our patients and families. You may be asked to participate in other research studies in the future. You have the right to decide to participate or decline to participate in any future studies. We will not share your contact information with researchers outside Nationwide Children’s Hospital.

The method used in this clinical trial, systemic delivery of the micro-dystrophin gene using AAV, was invented at Nationwide Children’s Hospital (“Invention”). The Principal Investigator, Dr. Jerry Mendell, is one of the inventors. Sarepta is a publicly traded company. Sarepta has paid money to Nationwide Children’s Hospital for the option to license the Invention. Part of the money paid to Nationwide Children’s Hospital has been disbursed to Dr. Mendell.

Nationwide Children’s Hospital may receive future payments and other compensation from Sarepta based on data from this research study and/or if the experimental treatment is approved by the FDA and sold in the marketplace. Thus, Nationwide Children’s Hospital has a financial interest in the outcome of this study.

Dr. Mendell will not be receiving future payments from Sarepta related to the outcome of this research study.

Linda Lowes and Lindsay Alfano are physical therapists who work on this study, and are also consultants for Sarepta on their research studies. They do not personally receive payment for these consulting services. Sarepta is paying the Research Institute at Nationwide Children's Hospital for Linda and Lindsay’s work. The money will be used by the Research Institute to support other research. Linda Lowes’ and Lindsay Alfano’s consulting agreement with Sarepta has been reviewed and approved by Nationwide Children's Hospital as required by hospital policy.

Dr. Anne Connolly served on the Sarepta advisory board for the development of therapies for boys with DMD. Dr. Connolly received payment for these services.

Dr. Sahenk has no financial interest tied to the outcome of this Study. Dr. Sahenk will be receiving other payments from Sarepta for other inventions that are not related to nor used in this Study.

**13) HOW WILL MY STUDY INFORMATION BE KEPT PRIVATE?**

Information collected for this study includes information that can identify you. This is called “protected health information” or PHI. By agreeing to be in this study, you are giving permission to this study team to collect, use, and disclose your PHI for this research study and for future research purposes (including purposes that are currently unknown) unless otherwise allowed by applicable laws. Information collected is the property of Nationwide Children’s Hospital, its affiliated entities, and/or the sponsor.

Some of the information collected as part of this study will be sensitive, such as information relating to your genetic testing. This sensitive information may be used or disclosed for future unknown

research purposes.

PHI that may be used or disclosed will include:

• Information such as names, addresses, telephone/fax numbers and e-mail addresses

• Dates such as admission/discharge and birth/death

• Identifiers such as finger or voice prints or photographic images

• Identifying numbers, characteristics or codes, medical records,

• Any other unique identifying number, characteristic, or code

Demographic information that includes name, address and birth date is collected for registration purposes and to determine if you are eligible to be in the study. Admission and discharge dates are collected to measure study endpoints. Your medical records number is collected to review your medical history and to continue to collect information about your conditions during the study.

**People or Companies authorized to use, disclose, and receive PHI collected or created by this research study:**

• PI and study staff

• Other health care professionals (such as doctors) or providers (such as hospitals) that may provide services to you related to the study (for example, if you are injured)

• The Nationwide Children’s Hospital Institutional Review Board (the committee that reviews

all human subject research)

• Nationwide Children’s Hospital internal auditors

• Sarepta Therapeutics and its representatives (including outside laboratories working on this study, monitors, and auditors)

• The Food and Drug Administration (FDA), Office for Human Research Protections (OHRP), National Institute of Health (NIH), and other regulatory agencies within and outside the United States

• Your insurance company (if charges are billed to insurance)

Because of the need to give information to these people, absolute confidentiality cannot be guaranteed. Information given to these people may be further disclosed by them and no longer be protected by federal privacy rules.

**Reason(s) why the use or disclosure is being made:**

Your PHI could be used and disclosed for the following purposes:

• To conduct the research study and related activities

• To confirm the accuracy of the data collected as part of the research study

• To monitor that the study is carried out in accordance with the law and recognized standards for conducting research studies

• To seek approval from government agencies to market the study drug

• To undertake future research (using study data or specimens already collected)

• To comply with legal requirements

• To allow healthcare providers to provide medical care, obtain payment for the care, and conduct routine operations

A third-party organization, called Colpitts World Travel, has been contracted by the study sponsor to provide travel and reimbursement coverage for study participants. The research coordinator at the clinical site will provide your information (full name, home address, phone/cell number and/or email address, date of birth and bank account details) to Colpitts to complete the travel and reimbursement coverage.

The research coordinator will then submit a travel request to the Colpitts team via the online portal to arrange travel to the study site. All personal information that you provide for this purpose will be kept completely confidential and it will not be shared by Colpitts with any third party, except as necessary to provide the service. Colpitts will not sell or provide your information to anyone else, including the study sponsor or their representatives, and will not contact you, except as necessary for arranging the visits.

Also, if all information that does or can identify you is removed from your personal health information, the remaining information will no longer be subject to this authorization and may be used or disclosed to others.

You may decide not to authorize the use and disclosure of your PHI. However, if you do not authorize the use and disclosure of your PHI, you will not be able to be in this study. If you agree to be in this study and later decide to withdraw, you may also withdraw your authorization to use your PHI. This request must be made in writing to the study doctor. If you withdraw your authorization, no new PHI may be collected and the PHI already collected may not be used unless it has already been used or is needed to complete the study analysis and reports. The sponsor may still use your personal information that was collected for the Sponsor before you withdrew your authorization. This

withdrawal will involve no penalty or loss of benefits to which you are otherwise entitled.

Please address this request to:

Jerry Mendell, MD

Nationwide Children’s Hospital

Clinical Research Services

700 Children’s Drive, T6B

Columbus, OH 43205

PHI will only be shared with the groups listed above, but if you have a bad outcome or adverse event from being in this study, the study team or other health care providers may need to look at your entire medical records.

The results from this study may be published but your identity will not be revealed.

The PHI collected or created under this research study will be used or disclosed as needed until the end of the study. The records of this study will be kept for an indefinite period of time and your authorization to use or disclose your PHI will not expire.

Even after the research study, you will generally not have access to information maintained separately in your research file for research purposes only

There is a risk that someone could get access to the information (data) we have collected about you. If those data suggested something serious about your health, it could be misused. For example, it could be used to make it harder for you to get or keep a job or insurance. The Genetic Information Nondiscrimination Act of 2008 (GINA) says that group and individual health insurers may not use your genetic information to determine whether you are eligible for insurance, how much you have to pay, nor can they request or require that you take a genetic test. We cannot guarantee that this will fully protect you. Also, this Federal law does not protect you against genetic discrimination by companies that sell life insurance, disability insurance, or long-term care insurance. Your privacy and the confidentiality of your data are very important to us. We will make every effort to protect them.

**14) USE OF INFORMATION FOR INFORMATION/SAMPLES FOR FUTURE RESEARCH USE**

Information that identifies you may be removed from your study data and any samples that are collected during this research study and your data and/or samples distributed to other investigators to be used for future research studies without your additional informed consent.

**Future Research Use of Identifiable Information:**

With your permission, we would like to store your identifiable information (including PHI) for future research purposes, and as part of such future research purposes, your identifiable information may be disclosed to people or entities not listed above, such as researchers not involved with this study, government agencies, research foundations, or pharmaceutical or device companies. This future research may or may not be related to your medical problem. This future research may include sensitive information. Any future research projects will be reviewed and approved by an Institutional Review Board which protects the rights, welfare, and safety of human research subjects. If your identifiable information including PHI is used or disclosed in future research studies, absolute confidentiality cannot be guaranteed. Information shared for future research may be shared further with others and no longer be protected by federal privacy rules.

If you decide at any time that you do not want your PHI stored for future research, you must make this request in writing to the Principal Investigator at:

Once we receive your written request, we will destroy your PHI. However, if we have already shared your PHI with another individual or entity, we will not be able to destroy any of the PHI that are no longer in our possession.

Nationwide Children’s Hospital retains the right to cease storage and destroy the PHI at any time without sending notice to you or obtaining your consent.

You do not have to agree to use of your PHI for future research in order to be in this study, and your

decision will not affect the care you receive from the study doctors or Nationwide Children’s Hospital.

I agree to allow my PHI to be stored and used for future research as described above: (initial)

YES NO

**Future Research Use of identifiable Samples:**

During the treatment and safety extension parts of the study, we will be collecting blood samples from you. Some of these samples may not be fully used so that there are leftover portions of the samples. The Sponsor would like to store the leftover blood, and biopsy- samples for research at a later time, perhaps even years from now. Blood and biopsy samples may be stored by the Sponsor up to 15 years. Storage could occur at the sponsor’s off site location. This future research may be for purposes other than those defined in this study. The samples and related information may be shared with other researchers at Nationwide Children’s Hospital or other researchers selected by the Sponsor to

perform research.

Use of your samples for future research may help researchers learn more about how to prevent, find, and treat your disease or condition. Your samples and related information may also be used in research for other diseases and conditions. Genetic material (such as DNA and RNA) may be removed from the stored samples and used for genetic testing.

Using your samples for future research will probably not help you, and you will not be told the results of any future research. Your doctor will also not be told the results of any future research. We do hope any research performed involving your samples and related information will help other people in the future.

Your samples and information will be used only for research and will not be sold. There is a possibility that future research may lead to development of products that will be sold to the public. If this happens, there is no plan to share any financial gain with you.

The results from this future research may be published but your identity will not be revealed.

If you decide at any time that you do not want your samples or related information stored for future research, you must make this request in writing to the study doctor at:

Jerry Mendell, MD

Nationwide Children’s Hospital

Clinical Research Services

700 Children’s Drive,

Columbus, OH 43205

Once we receive your written request, we will destroy your samples and related information. However, once your samples and related information have been de-identified, we will not be able to destroy them because we will not be able to link your samples or information back to you. Also, if we have already shared your samples or information with another individual or entity, we will not be able to destroy these samples or information that are no longer in our possession.

Nationwide Children’s Hospital may stop storing your samples and related information at any time. The samples or related information may be destroyed without sending notice to you or obtaining your consent.

You do not have to agree to use of your samples or related information for future research in order to be in this study. Your decision will not affect the care you will receive from the study doctors or Nationwide Children’s Hospital.

I agree to allow my samples and related information to be stored and used for future research as described above: (initial your choice)

YES NO

**15) VIDEO SHARING**

I agree that the Sponsor can ask me on a separate consent form, via my Study Doctor, if I agree for my videos from this study to be shared with others, like investors, analysts, and sponsor agents, and at scientific meetings: (initial your choice)

YES NO

**16) WHOM SHOULD I CALL IF I HAVE QUESTIONS OR PROBLEMS?**

If you have questions, concerns, or complaints about anything while on this study or you have been injured by the research, you have 24 hour access to talk to Dr. Jerry Mendell at 614-843-4496You can also contact the study coordinator at (614) 355-2765, during business hours.

If you have questions, concerns, or complaints about the research; if you have questions about your rights as a research volunteer; if you cannot reach the Principal Investigator; or if you want to call someone else, call (614) 722-2708, Nationwide Children's Hospital Institutional Review Board, (the committee that reviews all research involving human subjects at Nationwide Children’s Hospital).

**Signature Block for Children N/A, Adult Subject**

Your signature documents your permission for the named child to take part in this research.

Printed name of child

Signature of parent or individual legally authorized to consent Date & Time AM/PM

to the child’s general medical care

Printed name of parent or individual legally authorized to consent

to the child’s general medical care

Note: Investigators are to ensure that individuals who are not parents can demonstrate their legal authority to

consent to the child’s general medical care. Contact Legal Services if any questions arise.

Signature of second parent or individual legally authorized to Date & Time AM/PM

consent to the child’s general medical care

Printed name of second parent or individual legally authorized to consent to the child’s general medical care

If signature of second parent not obtained, indicate why: (select one)

❑ Not required by IRB

❑ Second parent is deceased

❑ Second parent is unknown

❑ Second parent is incompetent

❑ Second parent is not reasonably available

❑ Only one parent has legal responsibility for the care and custody of the child

Signature of person obtaining consent Date & Time AM/PM

Printed name of person obtaining consent

**Assent**

Signature of subject Date & Time AM/PM

❑ Not obtained because the capability of the subject is so limited that the subject cannot reasonably be consulted.

**N/A, Witness not required**

My signature below documents that the information in the consent document and any other written information was accurately explained to, and apparently understood by, the subject, and that consent was freely given by the subject.

Signature of witness to consent process Date & Time AM/PM

Printed name of person witnessing consent process

**Signature Block for Adult Participation N/A, Pediatric Subject**

Your signature documents your permission to take part in this research.

Signature of subject Date & Time AM/PM

Printed name of subject

Signature of person obtaining consent Date & Time AM/PM

Printed name of person obtaining consent

**N/A, Witness not required**

My signature below documents that the information in the consent document and any other written information was accurately explained to, and apparently understood by, the subject, and that consent was freely given by the subject.

Signature of witness to consent process Date & Time AM/PM

Printed name of person witnessing consent process

**Signature Block for Adult Unable to Consent N/A**

Your signature documents your permission for the named person to take part in this research.

Printed name of subject

Signature of legally authorized representative Date & Time AM/PM

Printed name of legally authorized representative

| Signature of person obtaining consent |  | Date & Time | AM/PM |
| --- | --- | --- | --- |
| Printed name of person obtaining consent  **Assent** |  |  |  |
| Signature of subject |  | Date & Time | AM/PM |

❑ Not obtained because the capability of the subject is so limited that the subject cannot reasonably be consulted.

**N/A, Witness not required**

My signature below documents that the information in the consent document and any other written information was accurately explained to, and apparently understood by, the subject, and that consent was freely given by the subject.

Signature of witness to consent process Date & Time AM/PM

Printed name of person witnessing consent process

x x x

| **STUDY TIMELINE** | | | | | | | | | | | | | | | | | | | | | | | | |
| --- | --- | --- | --- | --- | --- | --- | --- | --- | --- | --- | --- | --- | --- | --- | --- | --- | --- | --- | --- | --- | --- | --- | --- | --- |
| **Study Interval** | **Bl** | **Vector Infusion** | | | **Follow Up** | | | | | | | | | | | | | | | | | | | |
|  | **Scr** | **(Inpatient)** | | |  |  |  |  |  |  |  |  |  |  |  |  |  |  |  |  |  |  |  |  |
| **Visit #** | **1** | **2** | | | **3** | **4** | **5** | **6** | **7** | **Opt** | **8** | **9** | **10** | **11** | **12** | **13** | **14** | | **15** | **16** | | **17** | **18** | |
| **Study Interval** | **-60** | **-1 d** | **0 d** | **1 d** | **7 d** | **14 d** | **30 d** | **60 d** | **90 d** | **1-2 w** | **180 d** | **9 m** | **1 y** | **18 m** | **2 y** | **30 m** | **3 y** | | **42 m** | **4 y** | | **54 m** | **5 y** | |
|  | **to -2** |  |  |  |  |  |  |  |  | **post** |  |  |  |  |  |  |  |  |  |  |  |  |  |  |
|  |  |  |  |  |  |  |  |  |  | **biopsy** |  |  |  |  |  |  |  |  |  |  |  |  |  |  |
| **Visit Window (days)** |  |  |  |  | **±7** | **±7** | **±7** | **±14** | **±14** | **±14** | **±14** | **±14** | **±14** | **±21** | **±21** | **±21** | **±21** | | **±21** | **±21** | | **±21** | **±21** | |
| Informed Consent | x |  |  |  |  |  |  |  |  |  |  |  |  |  |  |  |  |  |  |  |  |  |  |  |
| Medical History | x |  |  |  |  |  |  |  |  |  |  |  |  |  |  |  |  |  |  |  |  |  |  |  |
| Vitals | x | x | x | x | x | x | x | x | x | x | x | x | x | x | x | x | x | | x | x | | x | x | |
| Physical Exam | x | x |  | x | x | x | x | x | x | x | x | x | x | x | x | x | x | | x | x | | x | x | |
| ECHO/ECG | x |  |  |  |  |  | x |  |  |  |  |  | x |  | x |  |  |  |  |  |  |  |  |  |
| Chest X-Ray | x |  |  |  |  |  |  |  |  |  |  |  |  |  |  |  |  |  |  |  |  |  |  |  |
| MRI a | x |  |  |  |  |  |  |  |  |  | x |  | x |  |  |  |  |  |  |  |  |  |  |  |
| Hepatitis B & C, HIV | x |  |  |  |  |  |  |  |  |  |  |  |  |  |  |  |  |  |  |  |  |  |  |  |
| Biomarker Testing | x | x |  | x | x | x | x | x | x | x | x | x | x | x | x | x | x | | x | x | | x | x | |
| Safety Labsb | x | x |  | x | x | x | x | x | x | x | x | x | x | x | x | x | x | | x | x | | x | x | |
| Urinalysis | x | x |  | x | x | x | x | x | x | x | x | x | x | x | x | x | x | | x | x | | x | x | |
| Physical Therapy | x |  |  |  |  |  | x | x | x | x | x | x | x | x | x | x | x | | x | x | | x | x | |
| Assessments |  |  |  |  |  |  |  |  |  |  |  |  |  |  |  |  |  |  |  |  |  |  |  |  |
| Gene Transfer |  |  | x |  |  |  |  |  |  |  |  |  |  |  |  |  |  |  |  |  |  |  |  |  |
| Immunology Studies | x |  |  |  | x | x | x | x | x |  | x | x | x | x | x | x | x | | x | x | | x | x | |
| Muscle Biopsy | x |  |  |  |  |  |  |  | x |  |  |  |  |  |  |  |  |  |  |  |  |  |  |  |
| Photograph of Injection | x | x | x | x | x | x | x |  |  |  |  |  |  |  |  |  |  |  |  |  |  |  |  |  |
| Site |  |  |  |  |  |  |  |  |  |  |  |  |  |  |  |  |  |  |  |  |  |  |  |  |
| Adverse Events | x | x | x | x | x | x | x | x | x | x | x | x | x | x | x | x | x | | x | x | | x | x | |
| Concomitant Medications | To be collected from time of consent until final study visit, recorded on separate CRF | | | | | | | | | | | | | | | | | | | | | | | |

**STUDY TIMELINE**

ALT=alanine aminotransferase; AST=aspartate aminotransferase; Bl=baseline; BUN=blood urea nitrogen; CBC=complete blood count; CK=creatine kinase; CRF=case report form; d=day; ECG=electrocardiogram; ECHO=echocardiogram; GGT=gamma-glutamyl transferase; HIV=human immunodeficiency virus; INR=international normalized ratio; m=month; MRI=magnetic resonance imaging; Opt=optional; PT=prothrombin; PTT=partial thromboplastin time; Scr=screening; y=year.

a MRIs will only be performed on subjects greater than 3 years of age (Cohort B) at the time of enrollment. Only conscious sedation will be used during the cardiac MRI. If general anesthesia is required, the cardiac MRI will not be performed.

bSafety labs (CBC/Diff/Platelet with smear, PT/PTT/INR, Electrolytes, ALT, AST, Alkaline Phosphatase, Amylase, BUN, CK (preferably on 2 day visits but may be tested on a one-day visit per PI discretion. Creatinine, Cystatin

C, GGT, Glucose, Total Protein, Total Bilirubin, Urinalysis)
